# Scheduling allocation in 5G slicing networks utilizing weighted exponential and logarithmic functions to improve QoS

**DOI:** 10.1038/s41598-025-17385-4

**Published:** 2025-09-01

**Authors:** Xiaofeng Nong, Xiaobo Liang

**Affiliations:** 1 Network and Information Center, Guilin Tourism University, Guilin, 541000 Guangxi China; 2 Network and Educational Technology Center, Guilin Normal College, Guilin, 541000 Guangxi China

**Keywords:** 5G scheduling algorithm, Radio resource allocation, Live broadcast traffic, Weighted combination of exponential-logarithmic rules (HWEL), Quality of service, 5G slicing networks, Engineering, Mathematics and computing

## Abstract

Offering media-rich services, such as streaming videos, for emergency services requires compliance with reliability standards. The deployment of fifth-generation (5G) networks enables a wide range of services and applications with diverse Quality of Service (QoS) requirements. Supporting heterogeneous performance and migrating vital services to 5G networks pose significant challenges for emergency service providers in maintaining QoS. To address this, schedulers allocate resources to various traffic types in a QoS- and channel state-aware manner. The exponential function scheduling method (EXP RULE) is a well-established approach; however, it requires optimization to reduce packet loss rate and latency. This study proposes the Hybrid Weighted Exponential and Logarithmic Rule (HWEL RULE), which enhances EXP RULE by integrating weighted logarithmic functions to improve QoS metrics in 5G slicing networks. Operating within a Fog Radio Access Network (F-RAN) framework with Network Functions Virtualization (NFV), HWEL RULE dynamically allocates Baseband Unit (BBU) resources to Ultra-Reliable Low-Latency Communications (URLLC), Enhanced Mobile Broadband (eMBB), and Massive Machine-Type Communications (mMTC). Using LTE-Sim simulations, HWEL RULE demonstrates up to a 30.06% reduction in packet loss rate, 21% lower latency for video traffic, 23.5% lower latency for VoIP, 8.6% higher throughput, and 1.2% improved fairness compared to EXP RULE. This incremental enhancement ensures compatibility with existing 5G architectures while significantly improving real-time traffic performance.

## Introduction

Thanks to technology that offer cutting-edge services, applications, and smart devices, our daily lives are much more convenient^1,2^. The need for communication services is growing as new technology and applications are developed. Communication systems will handle this enormous growth in services and applications with 5G and beyond^3^. These applications and services call for more efficient networks with higher data rates, lower latency, more energy, more spectral power, and more network capacity^4,5^. The goal of the fifth generation of 5G mobile systems is to improve consumers’ perceived performance, or Quality of Experience (QoE), and to deliver faster data speeds than the prior order of 4G gigabit per second^6^. QoE is a metric that evaluates the end user’s happiness with network services from a user experience standpoint, including factors like video streaming quality, and is affected by metrics such as latency and throughput. Three primary categories of 5G applications have been established by the International Telecommunication Union (IUT): MTC, URLLC, and mobile broadband (eMBB)^7–9^.

The 5G radio access network (RAN), fronthaul, backhaul, and core network will all be improved as part of the International Telecommunication Union’s (ITU) goal for 5G networks in order to facilitate global connectivity, enable a variety of business cases, and build an agile and adaptable network^10^. Therefore, in addition to its enormous contribution to raising the speed and bandwidth of current 5G cellular networks, the 5G network represents various enhancements^11^. The new 5G architecture is made to use a few promising technologies, such as NFV and F-RAN, in order to meet the criteria and objectives of 5G. The primary technologies that make 5G networks possible are these two^12^. 5G networks necessitate dynamic resource allocation to accommodate a variety of applications, including industrial ones. Consequently, dynamic network functionalities for industrial applications in 5G have been examined^13^. Expanding 4G services to satisfy end-user demand for more mobile bandwidth is known as enhanced mobile broadband, or eMBB. To extend service areas, offer high data rate applications, offer smooth transmission for highway users, and uphold QoS standards in extremely crowded scenarios. It provides a plethora of services and applications, including cloud computing, video streaming, and augmented and virtual reality (AR/VR)^14–16^.

However, the use case of URLLC guarantees communication for vital applications like telemedicine, automated manufacturing, V2X communications, self-driving cars, and robots that demand low packet loss and low latency. It is difficult to satisfy URLLC QoS requirements due to the conflicting expectations of low latency and high reliability, as stated in^17^.

According to the strengths of 5G networks that were mentioned, the fastest way for consumers to benefit from this generation is through the faster transmission of voice, image, and data. We are having difficulties in this network in spite of these qualities^18^. One of these difficulties is ensuring quality of service in these kinds of networks. In this case, we need to create algorithms that offer the greatest service quality in the network because of the large number of users and their diverse behavior, which in turn leads to an increase in the diversity of traffic in the network^19,20^. One of the key elements in achieving satisfaction is offering the network’s proper service quality^21^. In order to improve key service quality metrics like latency, packet loss rate, operational capacity, and fairness, as well as to create an environment that will make users of these networks happier, this article aims to manage radio resources as much as possible.

The LTE network’s management of radio resources is referred to as packet scheduling. The eNodeB node, the radio base station in LTE networks, is where this operation occurs on the media access control (MAC) layer. All media access control, radio resource management, and radio communication control operations are housed within this node^22^. The majority of crucial operations, including scheduling requests, random access protocols, demultiplexing, and multiplexing, are carried out at the MAC layer. Appropriate scheduling algorithms on both the sending and receiving sides of the eNodeB are what improve the quality of service in the LTE access network. Any scheduling algorithm’s primary goal is to boost network performance and ensure equitable bandwidth distribution among users^23,24^.

This study focuses on channel state-aware and quality-of-service scheduling in 5G slicing networks, which are designed to meet the heterogeneous conditions of wireless environments, because of the wireless nature of 5G networks. “Network slicing” denotes the partitioning of 5G network resources into autonomous virtual segments, each tailored to facilitate a particular service type (such as live video or low-latency communications) to enhance service quality. The EXP RULE is the best scheduling technique that researchers in this field have introduced to date, based on experiments conducted. This scheduler creates the best possible schedule for every kind of traffic based on the state of the network. Compared to other packet scheduling techniques like M-LWDF, EXP/PF, and LOG RULE approaches, it has therefore greatly improved the service quality parameters and resulted in a decrease in the latency and packet loss rate^25^. But more work needs to be done to improve the quality of service provided by this technology, particularly with regard to the parameters of packet loss rate and delay for live broadcast traffic.

Therefore, utilizing the entire 5G network capacity for various live streaming traffic slices may become a standard practice in the future. To improve resource efficiency and preserve the QoS parameters needed for 5G network slices at the 5G F-RAN level, while preserving the separation of slices, the focus in this respect is on building a 5G F-RAN scheduler allocation. To ensure QoS of 5G F-RAN networks, an efficient packet-based scheduling sharing strategy is presented for 5G sliced live video streaming traffic with two operational modes.

To address the limitations of existing scheduling methods in 5G slicing networks, this study proposes the HWEL RULE, which enhances the EXP RULE by integrating weighted logarithmic functions. While EXP RULE effectively prioritizes real-time traffic, it exhibits shortcomings in packet loss rate and latency, particularly for URLLC and eMBB. HWEL RULE leverages a weighted combination of exponential and logarithmic metrics (with coefficients z = 0.9 and u = 0.1) within a F-RAN framework using NFV to dynamically allocate BBU resources. This approach supports diverse traffic types, including URLLC, eMBB, and mMTC, while maintaining compatibility with existing 5G architectures. The main contributions of this research are:

- Creating a 5G slice model to distribute F-RAN scheduling among traffic associated with various slices. According to this paradigm, a set of BBU resources is allocated to every kind of acceptable traffic.
- Creating a weighted combination scheduling approach using logarithmic and exponential functions, which uses unique coefficients to weight the connection and aid enhance service quality metrics.
- Improving VoIP and live video traffic quality of service parameters.
- Offering 5G video streaming traffic an efficient packet-based scheduling sharing scheme.
- The three cuts’ fairness in sharing resources for scheduling was examined and contrasted.

This study seeks to create an innovative scheduling algorithm named HWEL RULE to enhance the QoS metrics in 5G slicing networks. The primary aims of this study are: (a) to diminish latency and packet loss rates while enhancing throughput and fairness for real-time traffic, including video and VoIP; (b) to establish an F-RAN-based resource allocation model to accommodate various network slices with distinct requirements; and (c) to assess the performance of the proposed method against the existing EXP RULE method via simulation to evaluate the enhancements in QoS metrics. The rest of the article is organized as follows. “Related works” reviews the design of several scheduling methods that have been introduced by researchers so far. The network model is presented in “Network model”. In “Proposed method”, the formulation of the problem and the proposed method are reported. In  “Discussion and evaluation”, the comparison of the results of the proposed method with the exponential method has been evaluated from the point of view of service quality parameters. Finally,  “Conclusion” deals with conclusions and future works.

## Related works

Numerous research teams have created efficient and successful scheduling algorithms over time to raise the standard of service in mobile communication networks. The introduction of the fifth generation (5G) of mobile networks has brought attention to how crucial it is to use sophisticated planning methods in order to manage the restricted frequency spectrum that is available and still meet 5G transmission needs^26^. The scientific community has given this problem a lot of attention because of the growing need for mobile communications and the goal of having a completely linked society. Consequently, to address the demands of diverse applications and scenario scenarios, the scientific community has created new methodologies and a range of scheduling methods^27^. demonstrates that the optimal resource management problem for IoMT networks, constrained by simultaneous energy and load requirements, is NP-hard and exhibits significant time complexity. This issue has been analyzed. They propose a modular approach for energy and load management in the Internet of Medical Things (IoMT) utilizing the principles of network softwareization and virtual resources. The suggested controller dynamically allocates resources by precisely assessing the dimensions of the IoMT network. Subsequently, it allocates the load across IoMT servers and manages the traffic between switches to the designated server. Optimizing resource distribution in slicing networks represents a significant problem. For instance^28^, introduced an admission control model aimed at maximizing the long-term revenue of the network provider by the allocation of resources across diverse slices, highlighting the significance of this matter. Additionally, developing a deeper comprehension of advanced radio resource management will yield important knowledge for subsequent research and might be helpful for nascent scholars in the subject. The equitable distribution of resources among slices is crucial. A approach for online admission control prioritizing fairness in 5G/B5G networks is proposed in^29^, enhancing the equilibrium between efficiency and justice.

Enhancing wireless network QoE is a crucial problem. The majority of the accepted research is devoted to intracellular programming. They have demonstrated that higher throughputs and, for the most efficient, higher fairness are possible when opportunistic scheduling algorithms accommodate for channel disturbance. Enhanced infrastructures can optimize slicing performance. Addresses noise reduction in visible light positioning (VLP) systems, which can be related to interference challenges in 5G networks^30^. Advanced techniques, including reinforcement learning, have been evaluated for resource allocation in 5G. Paper^31^ employed reinforcement learning to allocate resources while addressing the varied requirements of users.

The study^32^ Coverage in wireless visual sensor networks has been investigated, which can be adapted to resource management in 5G networks. Moreover, NFV facilitates SDN by virtualizing diverse network devices and functions. The performance of MIMO antennas for V2V communications in 5G has been investigated, which helps to improve QoS in the 5G physical layer^33^. Furthermore, owing to the swift advancement of artificial intelligence, researchers are exploring the feasibility of integrating some AI methodologies into Software-Defined Networking (SDN) to enhance resource usage and overall performance. This review examines the SDN architecture and explores the load balancing issue inside SDN. Secondly, classifying AI-driven load balancing techniques and comprehensively assessing these mechanisms from various viewpoints.

In^34^, researchers introduce a low-power system named GreenVoIP for the resource management of cloud VoIP virtual centers. This system mitigates overload by regulating the quantity of VoIP servers and network devices, including switches, while concurrently promoting green computing through energy conservation. An efficient and privacy-preserving vertical federated learning system based on the XGBoost algorithm, ELXGB, is proposed in^11^, providing secure data alignment, training, and inference services.

Research^35^ Energy consumption optimization in IoT sensors with reinforcement learning has been investigated, which can help with resource allocation in 5G. A resource allocation protocol is implemented at the “Radio Access Network (RAN)” layer to enhance internal services and increase QoS in 5G. QoS tagging is a form of organizational communication enabling a local hub or egress station to indicate its neighbors for specific traffic modifications. QoS highlighting is beneficial for facilitating visitor adjustments. This paper^36^ offers a comprehensive analysis of AI-driven security methodologies, specifically focusing on machine learning and deep learning techniques, while evaluating their relevance and constraints within smart city contexts. They incorporate real-world case studies to exemplify successful tactics and emphasize issues need more investigation, particularly with emerging communication technology.

In^37^, researchers introduced Information dissemination in critical networks has been investigated, which can be adapted to real-time traffic management in 5G. Based on the channel quality indicator (CQI) derived from base station (BS) feedback and the RB allocation over time, the created weighting factor can ensure that edge users have equitable scheduling possibilities during the intra-sector scheduling process. The issue of growing packet loss rate (PLR) close to the delay threshold can be resolved in the interim by modifying the queuing delay and retransmission delay of HARQ packets. The results of the simulation demonstrate that the suggested algorithm performs better than the conventional scheduling algorithms.

## Network model

**Fig. 1:**
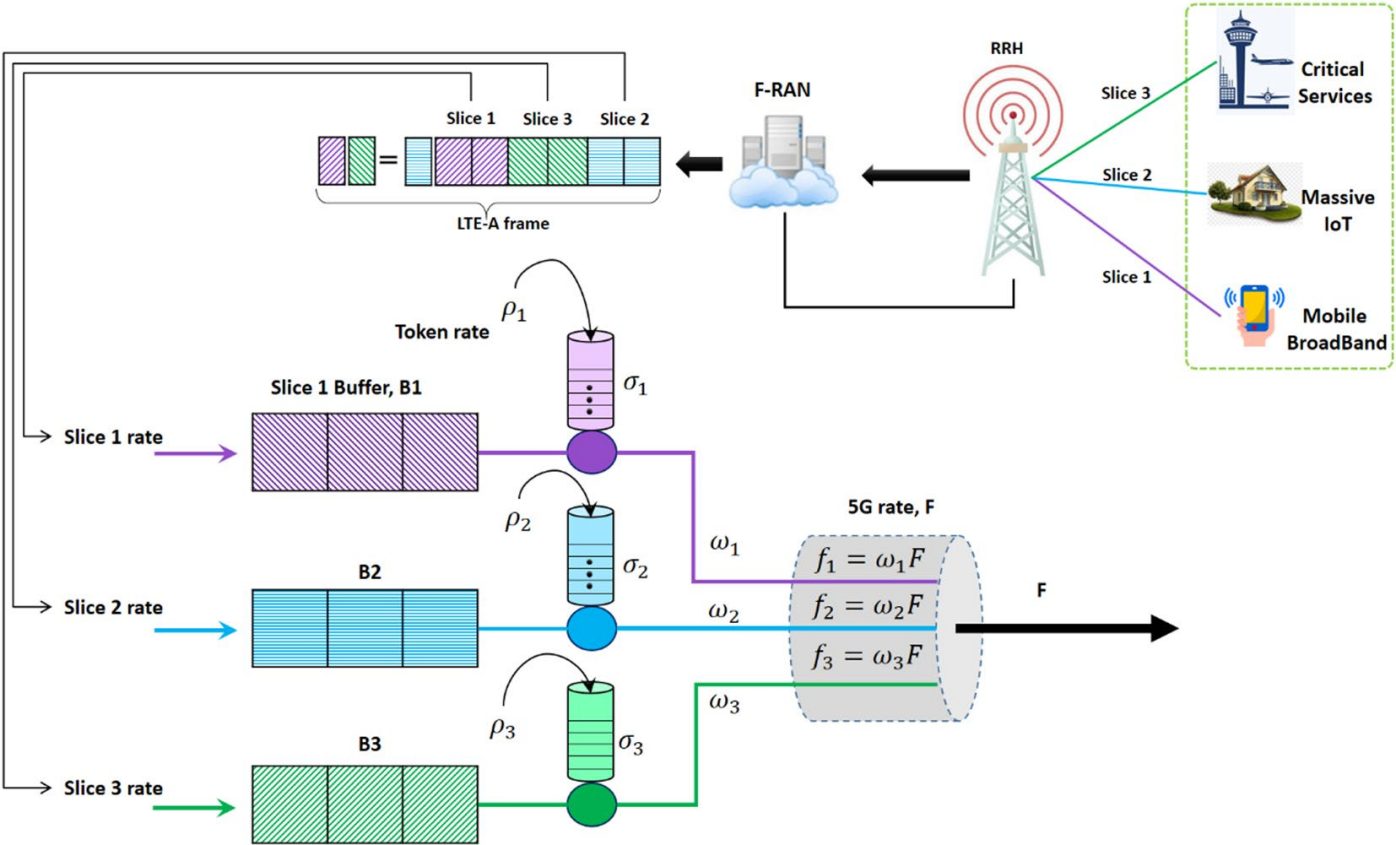
Scheduling model architecture based on the proposed method for different types of data traffic slices. The token rates $\:\left({\rho\:}_{i}\right)$ are set to accommodate the incoming traffic as follows: S1 (URLLC): $\:{\rho\:}_{1}=\:1.5\:Mbps$, S2 (eMBB): $\:{\rho\:}_{2}=\:3.0\:Mbps$, S3 (mMTC): $\:{\rho\:}_{3}=\:0.5\:Mbps$.

According to earlier research, a 5G NFV network infrastructure that was F-RAN enabled was taken into consideration. Figure 1 shows typical system components drawn. It was investigated how to allocate radio resources for data traffic in an F-RAN using a 5G slice. There are three different kinds of F-RAN architecture. For the purposes of this study, a completely centralized design with BBUs handling all network functions, such as medium access control (MAC), physical access control, and network layers, is assumed.

Working within the F-RAN, which is a component of network segmentation 5, was suggested an effective scheduling system based on two MAC layer modes of operation. Its job was to fairly distribute and manage the bandwidth allotted to each traffic segment.

According to the QoS requirements of the three main 5G slice types (URLLC, S1), Enhanced Mobile Broadband (eMBB, S2), and massive Machine-Type Communications (mMTC, S3) the token rates (ρ_i) in the leaky bucket regulator for each slice $\:i$ are determined in order to guarantee effective resource allocation in the suggested scheduling architecture for F-RAN networks. To accommodate each slice’s unique requirements, the token rates are set up as follows:

- S1 (URLLC): $\:\rho_1 \:=\:1.5\:Mbps$. designed to support high-priority, delay-sensitive traffic with stringent latency requirements.
- S2 (eMBB): $\:\rho_2 \:=\:3.0\:Mbps$. aimed at accommodating high-data-rate applications such as video streaming.
- S3 (mMTC): $\:\rho_3 \:=\:0.5\:Mbps$. tailored for low-rate, high-volume connections typical of Internet of Things (IoT) devices.

Fronthaul links are used to link RRH to the F-RAN. BBUs were the principal constituents of the BBU pool. BBU functions can be virtualized into many pieces known as virtualized BBUs (vBBUs) thanks to process virtualization, which is made possible by NFV in F-RAN. To buffer the packets of each 5G use case that the BBUs were waiting to handle, three sets of queues were linked to the BBU pool. Every queue was supplied with its own set of vBBUs in F-RAN. Each queue output was regulated by a leaky bucket regulator, and the queue size linked to the BBU was infinite. Lastly, we also considered the uniform distribution of user locations. Part of the transmission rate was assigned to each queued packet using the GPS-based mechanism that is part of the F-RAN.

At the F-RAN level, it is believed that a GPS-based mechanism controls a group of BBUs. There were N number of BBUs per set. The number of radio resources in BBUs can be measured in resource units or translated to a set of integer units to facilitate the analysis. The 5G frame version that was utilized determined how BBU resource units were defined. The LTE Advanced (LTE-A) specification is adhered to in this study, and BBUs are characterized as having a set of LTE-A Resource Blocks (RB). A BBU’s approved traffic segment was allocated to each set of RBs by the corresponding RRHs. Following the 5G frame time, each BBU resource’s transmission time was taken into account. A 5G LTE-A radio frame took up 12 ms, therefore it was made up of 22 slots of 0.4 ms each, plus a subframe that was made up of two time slots that were consecutive. The LTE radio frame consisted of 12 subframes altogether. The system’s entire transmission bandwidth determined the total number of RBs that were available. For instance, LTE with a 4 MHz bandwidth has 26 RBs, but when a 22 MHz bandwidth is used, it has 110 RBs. Each class received a different transfer rate dependent on the quantity of assigned resource units (RBs), with one RB serving as the minimum unit of allocation^38^.

For every transmission time slot that LTE-A defined, resource allocation and transmission were carried out. As a result, f was determined to be the F-RAN’s total output transmission rate expressed in bits per second. The total F-RAN throughput rate, denoted by F, is set to an average of 10 megabits per second (Mbps) for our simulated scenario, which is consistent with the system bandwidth of 5 MHz (corresponding to 25 RB) and the spectral efficiency calculated from the CQI.

### Traffic types and QoS requirements in 5G networks

To support the diverse needs of real-time applications in 5G networks, the proposed scheduling model considers three main traffic types defined by the International Telecommunication Union (ITU): URLLC, mMTC, and eMBB. Each traffic type has distinct characteristics that are relevant to specific QoS requirements and real-time applications.

Table 1 summarizes the key features, QoS requirements, demonstration examples, and allocated token rates for three major 5G traffic types URLLC, mMTC, and eMBB that are integrated into the proposed scheduling model. These parameters, including latency less than 1 ms and packet loss rate below 0.001% for URLLC (S1), latency up to a few seconds and packet loss rate below 1% for mMTC (S3), and latency less than 10 ms with high throughput for eMBB (S2), are aligned with ITU standards and reflect the diverse needs of real-time applications.

**Table 1:** Summary of traffic types and QoS Requirements.

Traffic type	Characteristics	QoS requirements	Examples	Token rate (Mbps)
URLLC (S1)	Mission-critical, low-latency, high-reliability	< 1 ms latency, < 0.001% packet loss rate	Telemedicine, autonomous vehicles	1.5
mMTC (S3)	Scalable, low-data-rate, high-connection volume	< 1% packet loss rate, latency up to seconds	Smart cities, agriculture	0.5
eMBB (S2)	High-data-rate, flexible latency	< 10 ms latency, < 1% packet loss rate, high throughput	4 K streaming, AR/VR	3.0

## Proposed method

The suggested 5G resource sharing scheduling strategy, which is anticipated to be utilized within an F-RAN entity of a 5G slice network, is presented and explained in this section. The proposed strategy is to maximize the consumption of F-RAN resources and maintain the minimum needed QoS for each 5G network segment while retaining its isolation. Each F-RAN entity in 5G networks has its own BBU resources. The proposed approach distributes BBU pool resources among three 5G segments with distinct traffic sources using the GPS model. Each traffic source distributes a resource segment based on the needed quality of service. Every segment in a 5G network has unique QoS requirements, including those related to delay and bandwidth^36^. As a result, the suggested approach essentially integrates the ideas of dynamic resource partitioning and GPS-based scheduling method in an F-RAN resource sharing method. In Fig. 1, the model and some of its components are displayed.

The operational phases’ framework and specific components are outlined in the sections that follow. These components are: a leaky bucket with token rate ρi and token size σi for each Bi, to establish the input rate of each slice; an infinite buffer for each slice i, Bi (i = 1,2,3); and a BBU reservoir that contains 5G Shared Resources for F-RAN The LTE-A frame structure defined the transmission time interval, which was followed by the implementation of the scheduling procedure at the 5G MAC layer level. Each slice’s cumulative data traffic arrival had a tail traffic distribution or followed a Poisson process. Each slice’s source inputs were shaped by a leaky bucket regulator to guarantee a maximum buffer size and traffic delay for each slice. Based on two settings, the leak bucket modifies each slice’s incoming traffic.

Each segment’s needed transmission bandwidth is translated into the total number of LTEs (available bandwidths), with a 185 kHz bandwidth assigned to each RB. As a result, at the start of each LTE-A slot time slot in the 5G F-RAN, the GPS-based scheduling system was in charge of assigning available RBs to each 5G slot type. Since the LTE-A frame structure defines the 5G system’s transmission time interval, the service time was therefore taken to be constant. The F-RAN system’s output transmission rate, denoted as C and expressed in bps, is contingent upon the system’s bandwidth. The three 5G segments were named S1 (uRLLC, or delay-sensitive traffic), S2 (mMBB, or heavy traffic), and S3 (mMTC, or popular traffic links). Each segment’s needed transmission rate for data traffic has a capacity rate (ci) that was predetermined based on its QoS criteria. Consequently, based on the three different types of 5G slices, the transmission rate capacity of F-RAN, C, was equal to the sum of the three segments. During transmission slot j of the LTE MAC frame, each segment, i, has a minimum capacity rate, ci(j) = wiC, where (i = 1, 2, 3) and wi denote the percentage of C given to each slice. Equation (1) defines the allowed data rate $\:{r}_{i,k}\left(t\right)$ for user iii at time ttt on the $\:{k}_{th}$ resource block. This rate is determined using spectral efficiency:

1 $$\:F>\sum\:_{\forall\:j}{r}_{i,k}\left(t\right)$$

The import of a slice’s packets marks the beginning of the suggested plan. If there is room, they are immediately placed into the buffer, Bi, that corresponds to the data traffic of that slice. The packages are taken out if not.

### Packet scheduling formulation

There are numerous ways to satisfy network user requests while maintaining the proper level of service quality. The ability to access radio sources for users to send and receive content is one of the key and fundamental solutions. Packet scheduling is the method used for this. Thus, scholars have created and presented a number of scheduling techniques. Determining each transmitted packet’s destination that is, the user to whom the packet should be sent is the goal of timing the packet in the communication channel to receive the signal. Additionally, it is decided from which common channel source block the transmission should be made for each of the chosen user(s). Federated learning explores removing low-quality data, which could help optimize data in managing 5G networks^39,40^.

A physical resource block (RB), which is defined in both the time and frequency domains, is the smallest radio resource unit that will be assigned to a user for data exchange in the communication channel for signal reception in the LTE network. The entire available bandwidth in the frequency domain is split up into 180 kHz subchannels, with each subchannel being equivalent to 12 consecutive subcarriers at a 15 kHz bandwidth (15 × 12 = 180 kHz). Time is split up into multiple frames in the time domain, with each frame having ten time intervals. It is required to clarify that each transmission has a time interval of one millisecond (ms) and consists of two time slots, each of which lasts for 0.5 ms. Additionally, seven OFDM signals (with a brief cyclic prefix) correlate to each time slot^5^.

Radio resources are allocated according to each time interval. A source block is a time or frequency radio source that consists of a subchannel (180 kHz) with 12 subcarriers in the frequency domain and a time slot of 0.5 milliseconds in the time domain. Resource block pairs are allocated to a user in the time domain for data exchange during each transmission interval^14^.

In LTE networks, the packet scheduling algorithm’s goal is to maximize system performance by distributing resources among users in an equitable and effective manner. Both live and non-live streaming traffic can be supported by a variety of scheduling techniques. The scheduling paradigm known as “independent of channel status” was initially developed for wired networks and operates under the premise that the channel is error-free and time-independent^40^. This kind of scheduling includes the RR, WFQ, EDF, LWDF, and FIFO approaches. While some of these methods have straightforward implementations, others offer the necessary service quality. Using SDN in the proposed model can improve the security of resource allocation. Therefore, a DoS attack mitigation scheme in SDN can enhance the security in such networks^41^. The scheduler estimates the channel quality for the users using the channel quality indicator, or CQI, which is transmitted to the eNodeB to learn the channel status from the users.

Channel state-aware or channel state-sensitive scheduling is the name given to this scheduling paradigm. This scheduling approach divides scheduling into two modes and two distinct goals. In the first mode or aim, it is crucial to give users the proper service quality; in the second mode or goal, it is crucial to distribute available bandwidth fairly among users. It should be mentioned that non-aware scheduling of service quality is the name of the second mode or objective. The MT, PF, M-LWDF, EXP/PF, EXP RULE, and LOG RULE methods can be noted as instances of channel state-aware scheduling. Only channel state-aware scheduling is carried out in LTE networks for live broadcast traffic, and it is based on user reports of the CQI indicator^21^.

The working principles of various scheduling algorithms that are aware of the channel status will be covered in this section, and the suggested approach will be offered at the end of the article. The definitions of terms used in calculations and relationships are also shown in Table 2.

**Table 2:** Expressions used in calculating scheduling metrics.

Abbreviation	Description
4G	Fifth generation
$\:{m}_{i\:,k}$	Generic metric for i-th user on k-th RB
$\:{d}_{k}^{i}\left(t\right)$	Acceptable data rate for i-th user at time t on k-th RB
CQI	Channel quality indicatior
EDF	Earliest deadline first
N	The number of users in the network
$\:\frac{\:\:\:\:\:\:\:\:\:\:\:}{\:{R}_{i}\left(t\right)}$	The average throughput obtained until time $\:t-1$
$\:{r}_{i,k}\left(t\right)$	Data rate received by user-i at time t
eNodeB	Evolved node B
EXP RULE	Exponential rule
EXP/PF	Exponential proportional fairness
$\:{\alpha\:}_{i}$	Weighting parameter
$\:{D}_{HOL,i}$	Head-of-line packet delay for each user
HWEL RULE	Hybrid weighted EXP LOG RULE
LOG RULE	Logarithmic rule
LTE	Long term evolution
$\:{N}_{rt}$	Number of live streaming traffic
LWDF	Largest weighted delay first
MAC	Media access control
M-LWDF	Modified- largest weighted delay first
$\:{{\Gamma\:}}_{k}^{i}$	The spectral efficiency of the channel obtained from the $\:-i$ user over the $\:-k$ RB
MT	Maximum throughput
OFDM	Orthogonal frequency division multiple
PF	Proportional fairness
RB	Resource block
RR	Round Robin
TTI	Transmission time interval
VoIP	Voice over internet protocol

#### Timing by MT method

This scheduling strategy distributes each RB to users with superior channel status, increasing system throughput. This implies that the user with the best channel status is given priority at all times. The system’s operational capacity rises while using this strategy. However, there will be a significant reduction in user fairness, and certain users who do not have a good channel status (for instance, they are at the edge of the cell) will not have their scheduling completed, which will cause them to starve. The size or value that is determined by any scheduling approach based on the quantity of radio resources and users for the pertinent traffic is called the metric. Equation (2) shows the MT technique metric^42^:

2 $$\:{m}_{i,k}^{MT}={d}_{k}^{i}\:\left(t\right)\:;1\le\:i\le\:$$

The permissible data rate for the *-i*_*th*_ user at time t on the *-k*_*th*_ RB is given by $\:{d}_{k}^{i}\:\left(t\right)$ in relation (1), where N is the total number of users in the network. Operational power is also computed using the SINR value [3 5]. Equation (3) illustrates how to compute $\:{d}_{k}^{i}\:\left(t\right)$:

3 $$\:{d}_{k}^{i}\:\left(t\right)=\text{log}\left[1+{{SINR}}_{k}^{i}\left(t\right)\:\right]\:$$

#### Timing by PF method

This technique strikes a compromise between user fairness and spectral efficiency. In this manner, a timetable for users without an appropriate channel status is also created. Equation (4) is used to derive the metric of this technique for the *-i*_*th*_ user on the *-k*_*th*_ RB:

4 $$\:{m}_{i,k}^{PF}=\frac{{d}_{k}^{i}\:\left(t\right)}{\overline{{R}_{i}(t-1)}\:\:}\:;1\le\:i\le\:N$$

where $\:\overline{{R}_{i}(t-1)}$, which comes from Eq. (5), is the average throughput up to time t-1:

5 $$\:\overline{{R}_{i}\left(t\right)}\:=\left(1-\frac{\text{1}}{\text{T}}\right)\overline{{R}_{i}(t-1)}+\frac{1}{T}{r}_{i}\left(t\right)$$

*T* is the fairness criteria window in this case, and its length is equal to TTI. The data rate that the *i-th* user got at time *t* is also represented by $\:{r}_{i}\left(t\right)$^43^.

#### Scheduling by M-LWDF method

This scheduler, which is used in wireless networks, is an extension of the LWDF scheduler^14^ and is cognizant of channel information. Different priorities are taken into account by this algorithm for broadcast traffic that is not live versus live broadcast traffic. This algorithm’s goal is to guarantee both a respectable degree of user fairness and a high system throughput. Equation (6) is used to obtain the metric of this approach.

6 $$\:{m}_{i,k}^{M-LWDF}={\alpha\:}_{i}{D}_{HOL,i}\frac{{d}_{k}^{i}\:\left(t\right)}{\overline{{R}_{i}(t-1)}\:\:}\:;1\le\:i\le\:N$$

The weighting parameter in relation (5) is $\:{\:\alpha\:}_{i}$, and the head-of-line packet delay for each user is $\:{D}_{HOL,i}$. According to theoretical research, the M-LWDF method’s fairness criterion is contingent upon several factors such as the channel’s condition, the user’s communication process, and the requirement for varying service quality levels for distinct types of traffic^44^. Theoretical research was done to enhance the fairness criterion in the M-LWDF scheduling method, and the result was the presentation of a modified approach based on the M-LWDF method that performs better than M-LWDF for live broadcast traffic on the communication channel side to receive the signal^45^.

#### Timing by EXP/PF method

For non-live broadcast traffic, this scheduler employs the PF approach; for live broadcast traffic, the delay is determined by an exponential function. This technique offers a decent degree of customer fairness while optimizing throughput and reducing latency for live streaming traffic. The EXP/PF time method’s metric is shown in relation (7):

7 $$\:{m}_{i,k}^{EXP/PF}=exp\left(\frac{{\alpha\:}_{i}{D}_{HOL,i}-{\stackrel{-}{D}}_{HOL,i}}{1+\sqrt{{\stackrel{-}{D}}_{HOL,i}}}\right)\frac{{d}_{k}^{i}\:\left(t\right)}{\overline{{R}_{i}(t-1)}\:\:}\:;1\le\:i\le\:N$$

Equation (8) is used in the following equation to determine the average head-of-line packet delay for each user $\:{\stackrel{-}{D}}_{HOL,i}$.

8 $$\:{\stackrel{-}{D}}_{HOL,i}=\frac{1}{{N}_{rt}\:\:}{\sum\:}_{i=1}^{\begin{array}{c}{N}_{rt}\end{array}}{\alpha\:}_{i}{D}_{HOL,i}\:$$

The number of live broadcast traffic on the side of the communication channel to receive the signal is equal to $\:{N}_{rt}$ in relation (8)^18^. In Eq. (8), the average header delay (HOL), denoted as $\:{D}_{HOL,i}$, is computed for each user and used into the scheduling criterion of the EXP/PF approach. This criterion is influenced by the T fairness window, used in the computation of the average throughput $\:{R}_{i}\left(t\right)$ as delineated in Eq. (5). The *T* parameter regulates the impact of historical throughput on the current data rate. An elevated *T* value results in a more gradual alteration of $\:{R}_{i}\left(t\right)$, so enhancing the priority of users experiencing greater delays (i.e., those with older HOL packets) and potentially augmenting $\:{D}_{HOL,i}$ for users with lower data rates, while simultaneously improving overall fairness. In contrast, a diminished *T* renders $\:{R}_{i}\left(t\right)$ more sensitive to immediate rate variations, thereby decreasing latency for users with elevated data rates, but at the expense of fairness, so privileging users with superior channel conditions, such as those in proximity to the base station. Consequently, the selection of *T* necessitates a compromise between equity and latency minimization and should be made judiciously, considering traffic attributes and network specifications.

#### Scheduling by LOG RULE method

By limiting the delay parameter, the logarithmic scheduling method (LOG RULE) offers network users a high level of service quality. This approach is a scheduling technique that balances quality of service in terms of delay and throughput criteria. It is aware of the channel condition and quality of service. Equation (9) is the basis for this method’s measurement and scheduling of all traffic kinds^46^:

9 $$\:{m}_{i,k}^{LOG-RULE}={b}_{i}\text{log}(1.1+{\alpha\:}_{i}{D}_{HOL,i}){{\Gamma\:}}_{k}^{i}\:$$

A metric called $\:{m}_{i,k}^{LOG-RULE}$ is computed for the *-i*_*th*_ user on the -kth Resource Block, taking into account the LOG RULE for all kinds of traffic. In Eq. (9), the parameters $\:{\alpha\:}_{i}$ and bi are crucial to the efficacy of the LOG RULE scheduling mechanism. The weight $\:{\alpha\:}_{i}$ enhances the scheduling priority of user *i*, potentially augmenting their throughput at the expense of greater delay for others. This modification is especially advantageous in delay-sensitive applications like URLLC, where minimizing latency is essential. Conversely, $\:{b}_{i}\:$regulates the sensitivity of the scheduling metric $\:{M}_{i,log}$ to fluctuations in throughput. A bigger $\:{b}_{i}$ results in a more stable measure, benefiting users with lesser throughput, hence enhancing fairness and minimizing delays in crowded network environments such as mMTC, although this comes at the cost of overall system performance. Conversely, a smaller $\:{b}_{i}$ emphasizes high-throughput consumers (e.g., in eMBB services), enhancing total throughput but potentially increasing latency for customers with suboptimal channel conditions, particularly under large video traffic loads. Consequently, meticulous adjustment of $\:{\alpha\:}_{i}$ and $\:{b}_{i}$ is crucial to satisfy the distinct demands of various traffic kinds and service contexts^47^. The computed metric is used to decide how radio resources should be distributed among various traffic kinds. Numerous variables, including $\:{\alpha\:}_{i}$ and $\:{b}_{i}$, can be changed in connection to (10). The spectral efficiency of the channel, which is derived from the *-i*_*th*_ user in the *-k*_*th*_ RB, is indicated by the parameter $\:{{\Gamma\:}}_{k}^{i}$, where $\:1\le\:i\le\:N$. In Eq. (10), $\:E\left[{{\Gamma\:}}^{i}\right]$ represents the average spectral efficiency of the channel for user iii. The inverse of this value is used in the calculation of the LOG RULE metric to improve fairness in resource allocation.

10 $$\:{\alpha\:}_{i}\in\:\left[\frac{5}{\left(0.99{\tau\:}_{i}\right)},\frac{10}{\left(0.99{\tau\:}_{i}\right)}\right]\:,{b}_{i}=\frac{1}{E\left[{{\Gamma\:}}^{i}\right]}\:$$

The closed waiting time threshold value, represented by$\:{\:\alpha\:}_{i}\:$in, is chosen from the set of values given in Eq. (10). The timing function is optimized with this decision, which explains why the coefficient of 0.99, rather than the coefficient of 1, is found in the denominator associated with the parameter $\:{b}_{i}$. Actually, a small adjustment to this choice will improve the accuracy of the metric coefficient used by the LOG-RULE scheduler. Additionally, the parameter $\:{b}_{i}$ in Eq. (10), which comes from the i-th user in the *k-th* RB, is the inverse of the mathematical expectation (average) of the channel’s spectral efficiency.

#### Scheduling by EXP RULE method

The enhanced EXP/PF technique and the EXP RULE are two scheduling strategies that are offered to ensure the service quality of users in a shared wireless link. They are based on the knowledge of channel state and quality of service. It is evident that this method takes queues and channel conditions into account while scheduling packets. The following formula for the time metric is computed exponentially^9^:

11 $$\:{m}_{i,k}^{EXP-RULE}={b}_{i}{exp}\left(\frac{{\alpha\:}_{i}{D}_{{HOL},i}}{1+\sqrt{\left(\frac{1}{{N}_{{rt}}{\:\:}}\right){\sum\:}_{j=1}^{\begin{array}{c}{N}_{{rt}}\end{array}}{D}_{{HOL},j}}}\right){{\Gamma\:}}_{k}^{i}\:\:\:\:\:$$

Similar to the logarithmic method, this approach has restrictions for the values $\:{\alpha\:}_{i}$ and $\:{b}_{i}$^19^. Equation (12) presents the values of these constants:

12 $$\:{\alpha\:}_{i}\in\:\left[\frac{5}{\left(0.99{\tau\:}_{i}\right)},\frac{10}{\left(0.99{\tau\:}_{i}\right)}\right]\:,{b}_{i}=\frac{1}{E\left[{{\Gamma\:}}^{i}\right]}\:\:$$

In Eq. (12), $\:E\left[{{\Gamma\:}}^{i}\right]$ is considered as the average spectral efficiency of the channel for user iii. This value is used in the EXP RULE metric to prioritize real-time traffic.

Exponential scheduling has enhanced system throughput, justice criteria, and delay parameters based on multiple measurements^16^. It should be noted that live broadcast traffic has higher priority than non-live traffic when using this strategy^20^. The disadvantage of this approach is that packet scheduling does not take user throughput into account at any given time. Additionally, this approach raises the rate of packet loss by not giving high priority to packets that are about to expire.

### Formulation of the proposed method

The goal of the weighted combination of exponential and logarithmic functions (HWEL RULE) scheduling approach is to outperform the exponential rule method in terms of quality of service characteristics, particularly delay, packet loss rate, and system throughput for live broadcast traffic.

The unused portion of the F-RAN transmission rate is dynamically shared using the weighted scheduling approach based on exponential functions. A precise transmission rate for all of the data traffic in each segment can be found using Eq. (5). The data traffic of other sectors that are operational and ready gets a fair share of the unused rate. At the beginning of each time slot, the transmission rate can be assigned as follows for each piece of data stream. We first assume that there are three slices, and that each slice i can either have buffered data at time j beginning with ri(j) ≠ 0 or it can have no buffered data to broadcast at time j beginning with ri (j) = 0. Thus, two crucial phases are involved in allocating the transmission rate. First, we compute U, the total transfer rate used by all slices containing buffered data, as follows:

13 $$\:U=\sum\:_{j\in\:rj\left(i\right)\ne\:0}{R}_{j}\left(i\right)={S}_{j}R,\:\:\:j=\text{1,2},3.$$

Second, the following formula is used to get the NU, or unused transfer rate: NU > 0 since NU = C-U; Next, the data traffic of additional segments with buffered data receives an equitable share of the overall unused transfer rate based on the following ratios:

14 $$\:{e}_{j}\left(i\right)=\frac{Vj}{\sum\:_{\forall\:j\in\:rj\left(i\right)\ne\:0}Vj}\times\:UN$$

This approach combines the logarithmic and exponential scheduling techniques, and its metric may be found using Eq. (15):

15 $$\:{m}_{i,k}^{Hybrid\:EXP-LOG}=z\left({m}_{i,k}^{EXP-RULE}\right)+u\left({m}_{i,k}^{LOG-RULE}\right)\:,where:z+u=10\:$$

The weight of the metrics of the exponential and logarithmic functions in the suggested method are represented, respectively, by the proper and constant coefficients z and u in the relationship above. Ten is the total of the two coefficients, z and u. In order to arrive at the optimal combined value of the functions, this number which is normalized as a percentage coefficient was calculated through a variety of simulations using various combinations.

The HWEL RULE method is designed to improve QoS metrics in 5G slice networks, especially for real-time traffic such as video and VoIP. It combines EXP RULE and LOG RULE to achieve an optimal balance between latency, packet loss rate, throughput, and fairness. The formulation of this method is based on a scheduling metric that is calculated for each packet in each slice i in resource block k at time t. The HWEL metric (M_HWEL) is defined as:

16 $$\:{M}_{HWEL,i,k}\left(t\right)=z.{M}_{EXP,i,k}\left(t\right)+u.{M}_{LOG,i,k}\left(t\right)$$

The exponential scheduling metric, denoted as $\:{M}_{EXP,i,k}\left(t\right)$ and defined in Eq. (11), is designed to reduce delay for real-time traffic by prioritizing packets with higher latency. In contrast, the logarithmic scheduling metric, $\:{M}_{LOG,i,k}\left(t\right)$, defined in Eq. (9), focuses on fairness and optimizing throughput for users experiencing varying channel conditions. Two fixed weighting coefficients, z and uuu, are used to balance the influence of the exponential and logarithmic metrics, respectively, with the condition that $\:z+u=1.$ Based on extensive simulations, the optimal values were determined to be $\:z=0.9\:and\:u\:=\:0.1$, emphasizing the exponential metric to effectively minimize delay and packet loss for real-time traffic.

The rationale for combining exponential and logarithmic functions in the HWEL RULE lies in their complementary strengths for addressing the diverse QoS requirements of 5G slicing networks. The exponential metric,

17 $$\:{M}_{EXP,i,k}\left(t\right)={b}_{i}\:exp\left(\frac{{\alpha\:}_{i}{D}_{HOL,i}}{1+\sqrt{\frac{1}{{N}_{rt}}{\sum\:}_{j=1}^{{N}_{rt}}{D}_{HOL,i}}}\right){{\Gamma\:}}_{k}^{i}$$

prioritizes packets with high head-of-line delay $\:{D}_{HOL,i}$, ensuring low latency for delay-sensitive traffic like URLLC and video streaming, which require latency below 150 ms per ITU G.114 standards. However, this aggressive prioritization can significantly increase packet loss rates for non-real-time streams - up to 40% more, it has been shown. In contrast, the logarithmic metric,

18 $$\:{M}_{LOG,i,k}\left(t\right)=\:{b}_{i}\:log\left(1.1\:+{\alpha\:}_{i}{D}_{HOL,i}\right){{\Gamma\:}}_{k}^{i}$$

promotes fairness by adjusting resource allocation based on spectral efficiency $\:{{\Gamma\:}}_{k}^{i}$, benefiting eMBB and mMTC traffic with variable channel conditions. HWEL RULE, defined as:

19 $$\:{M}_{HWEL,i,k}\left(t\right)=\:z\:.{M}_{EXP,i,k}\left(t\right)+\:u\:.{M}_{LOG,i,k}\left(t\right)$$,

with $\:z=0.9$ and $\:\:u=0.1$, balances these objectives. The high $\:z$ value emphasizes low latency for URLLC, while the non-zero $\:u$ ensures fairness for eMBB/mMTC, mitigating starvation of non-real-time flows. The weights are derived from analytical models of 5G traffic patterns, where URLLC demands high exponential weighting, and eMBB/mMTC require logarithmic contributions for throughput stability.

The Table 3 shows the values taken into account for the z and u coefficients in this combination to run several tests and get the best results:

**Table 3:** Values ​​considered for coefficients u z in the proposed method.

Combination of functions	z	$\:u$
9 to 1 combination	9	1
8 to 2 combination	8	2
7 to 3 combination	7	3
6 to 4 combination	6	4

The HWEL RULE method uses a weighted combination of exponential and logarithmic functions to calculate the M_HWEL scheduling metric, which offers several advantages for real-time traffic scheduling in 5G slicing networks. The exponential function helps reduce the latency and packet loss rate for delay-sensitive traffic such as URLLC and eMBB by prioritizing packets with high end-to-end delay. In contrast, the logarithmic function improves fairness for users with weaker channel conditions by adjusting the sensitivity to throughput changes.

The higher weight $\:z=0.9$ gives the exponential function more influence, because real-time traffic such as video and VoIP are sensitive to low latency, and the exponential function effectively prioritizes high-latency packets. The weight $\:u=0.1$ for the logarithmic function helps maintain fairness without compromising latency for latency-sensitive traffic.

**Fig. 2:**
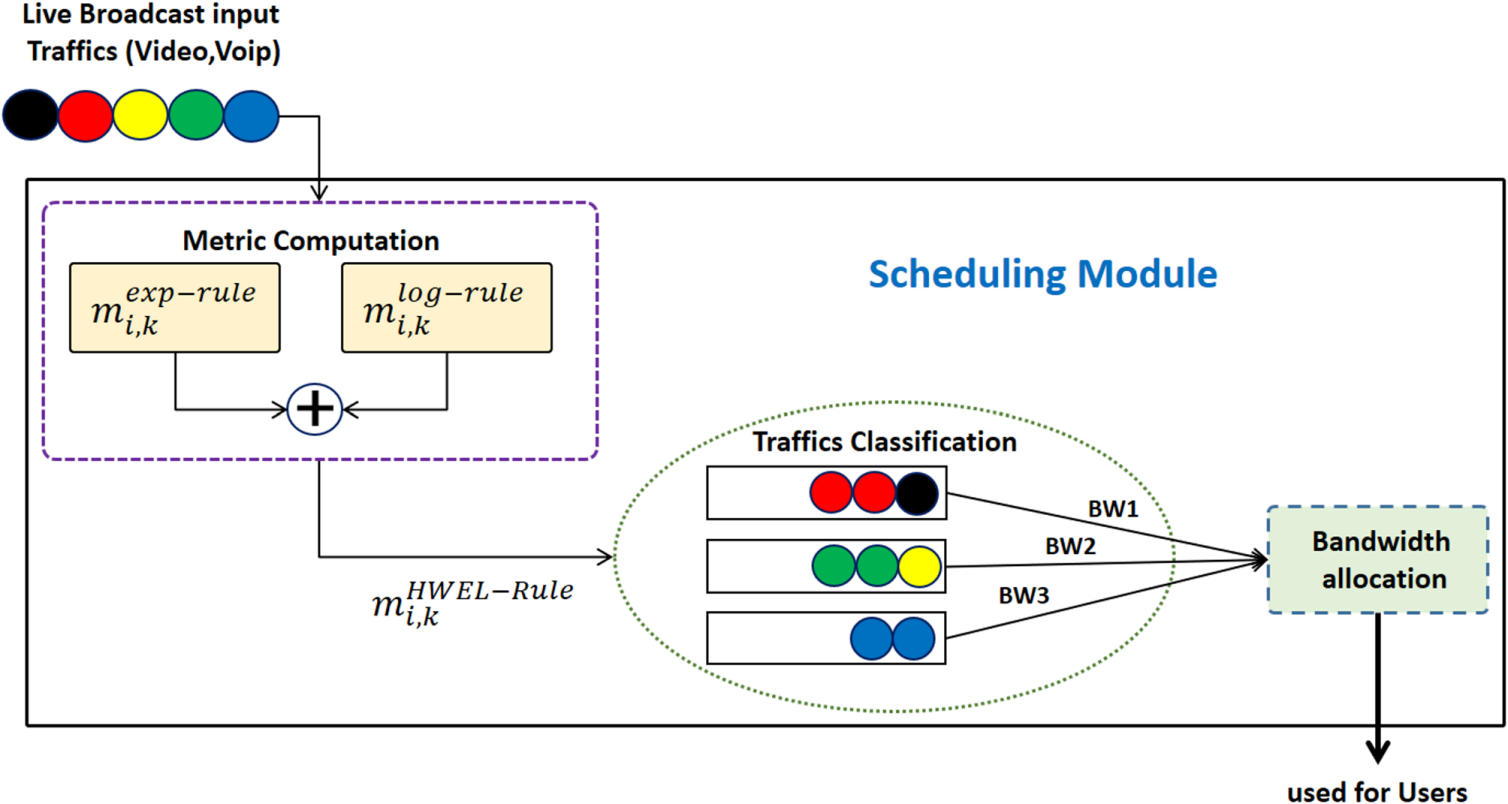
Using a weighted combination of logarithmic and exponential functions for scheduling with dynamic bandwidth allocation. Packets are grouped into three queues based on the M_HWEL metric and bandwidth $\:(BW1=0.7,\:BW2=0.2,\:BW3=0.1)$ is dynamically allocated based on traffic type and network conditions.

A design and setup schematic for this suggested method’s performance is shown in Fig. 2. This graphic illustrates how five distinct traffic packet types are thought to enter the scheduling module. This module first calculates the metrics linked to the logarithmic and exponential functions independently for each packet based on the status of the channel efficiency, latency, throughput, and packet loss rate. Next, as the last metric associated with the suggested strategy, $\:\left({m}_{i,k}^{HWEL-RULE}\right)$ is taken into consideration for each packet, which is the sum of two $\:\left({m}_{i,k}^{EXP-RULE}\right)+\left({m}_{i,k}^{LOG-RULE}\right)$. We will now group the packages into a number of queues based on the final metric that was determined for each package. Three queues are used as an example and hypothetically, meaning that just three queues are not employed in the simulation, in order to simplify the issue and shed light on the performance process of the scheduler created in this article in the form of a general model. As. Two threshold values, $\:m1$ and $\:m2$, where $\:m2$.

20 $$\:\begin{array}{c}{Q}_{1}\:if\:{m}_{i,k}^{HWEL-RULE}>m1\:\:\:\:\:\:\:\:\:\:\:\:\\\:{Q}_{2}\:if\:m2<{m}_{i,k}^{HWEL-RULE}<m1\\\:{Q}_{3}\:if\:{m}_{i,k}^{HWEL-RULE}<m2\:\:\:\:\:\:\:\:\:\:\:\end{array}$$

Rows $\:{Q}_{1}$, $\:{Q}_{2}$, and $\:{Q}_{3}$ are the first through third. Following categorization, bandwidth is allotted to each queue based on the kind of traffic that is present in it (BW1, BW2, and BW3). Bandwidth allocation in the proposed HWEL RULE method is performed dynamically and depends on the traffic conditions and QoS metrics in each time interval. As shown in Fig. 2, traffic packets are classified into three queues based on the M_HWEL metric, which is dynamically calculated from a weighted combination of exponential and logarithmic metrics. Bandwidth (BW1, BW2, BW3) is allocated to each queue based on the number of packets and the type of traffic present. In addition, the unused throughput (NU) in each time interval is fairly distributed among the queues with buffered data, which enhances this dynamic process. Then, every second, 100 packets 70 from the first queue, 20 from the second row, and 10 from the third leave the scheduler and are sent to users.

**Algorithm 1:**
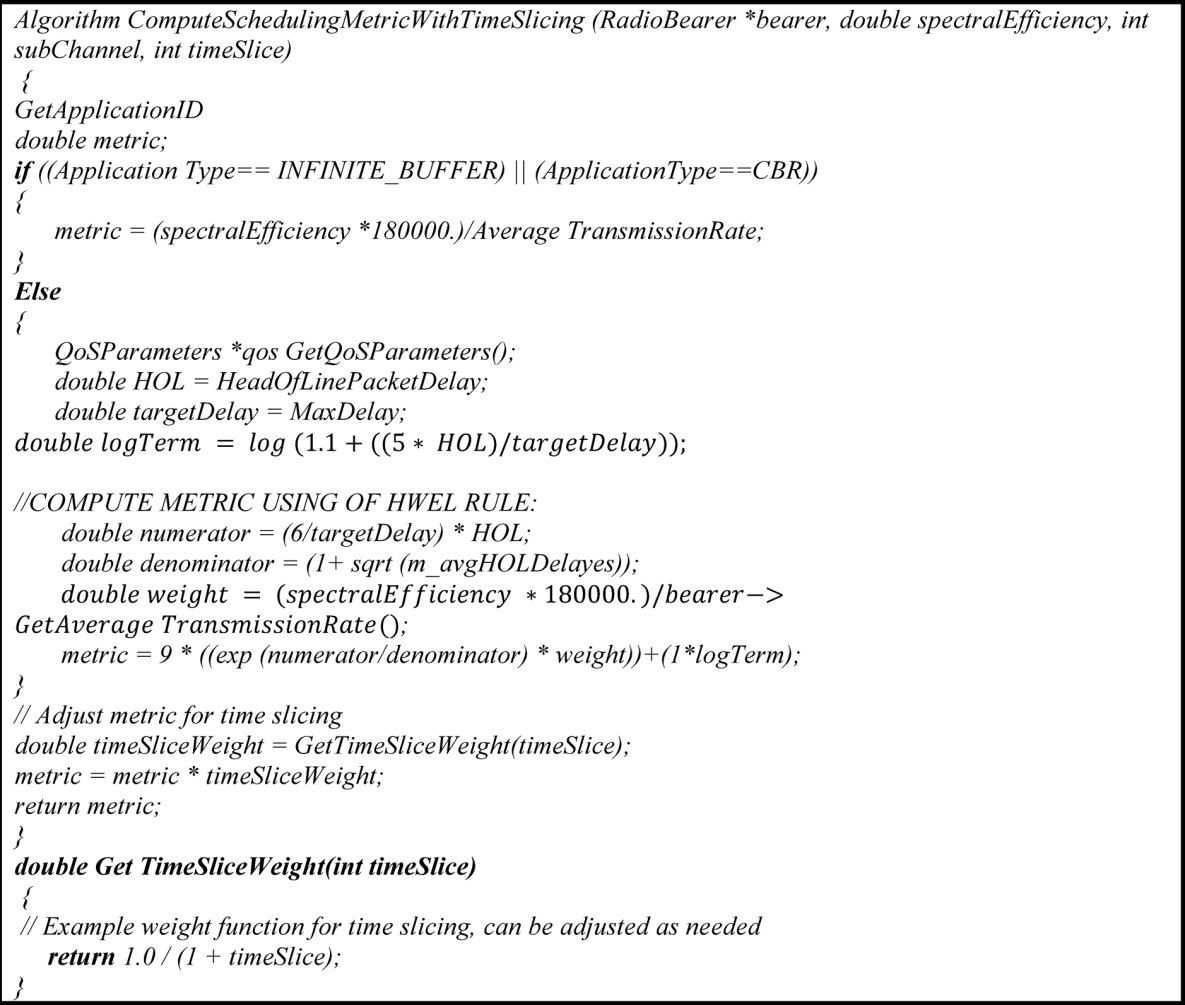
TimeSlice based on HWEL RULE.

Algorithm 1 implements a time-slicing-based scheduling method for radio resource allocation in 5G sliced ​​networks, which calculates a composite metric M_HWEL to prioritize traffic packets according to the QoS requirements of URLLC, eMBB, and mMTC slices. The algorithm first identifies the application type, such as infinite buffer or CBR, and for simple applications, calculates a metric based on spectral efficiency and average transmission rate. For QoS-sensitive traffic, the M_HWEL metric is composed of a 9:1 weighted combination of exponential and logarithmic metrics, which prioritize breakeven delay and fairness, respectively.

M_EXP uses the equation exp$\:\left(\left(\frac{6}{targetDelay}\right) \cdot \frac{HOL}{1+\sqrt{{m}_{avgHOLDelayes}}}\right)$ and spectral efficiency weighting to boost high-delay packets (such as URLLC), while M_LOG ensures fair resource distribution for mMTC with the form log(1.1+(5·HOL/targetDelay)). The final metric is adjusted by a time slice factor to optimize resource allocation in consecutive time slots, which is weighted by the function 1/(1+timeSlice).

**Fig. 3:**
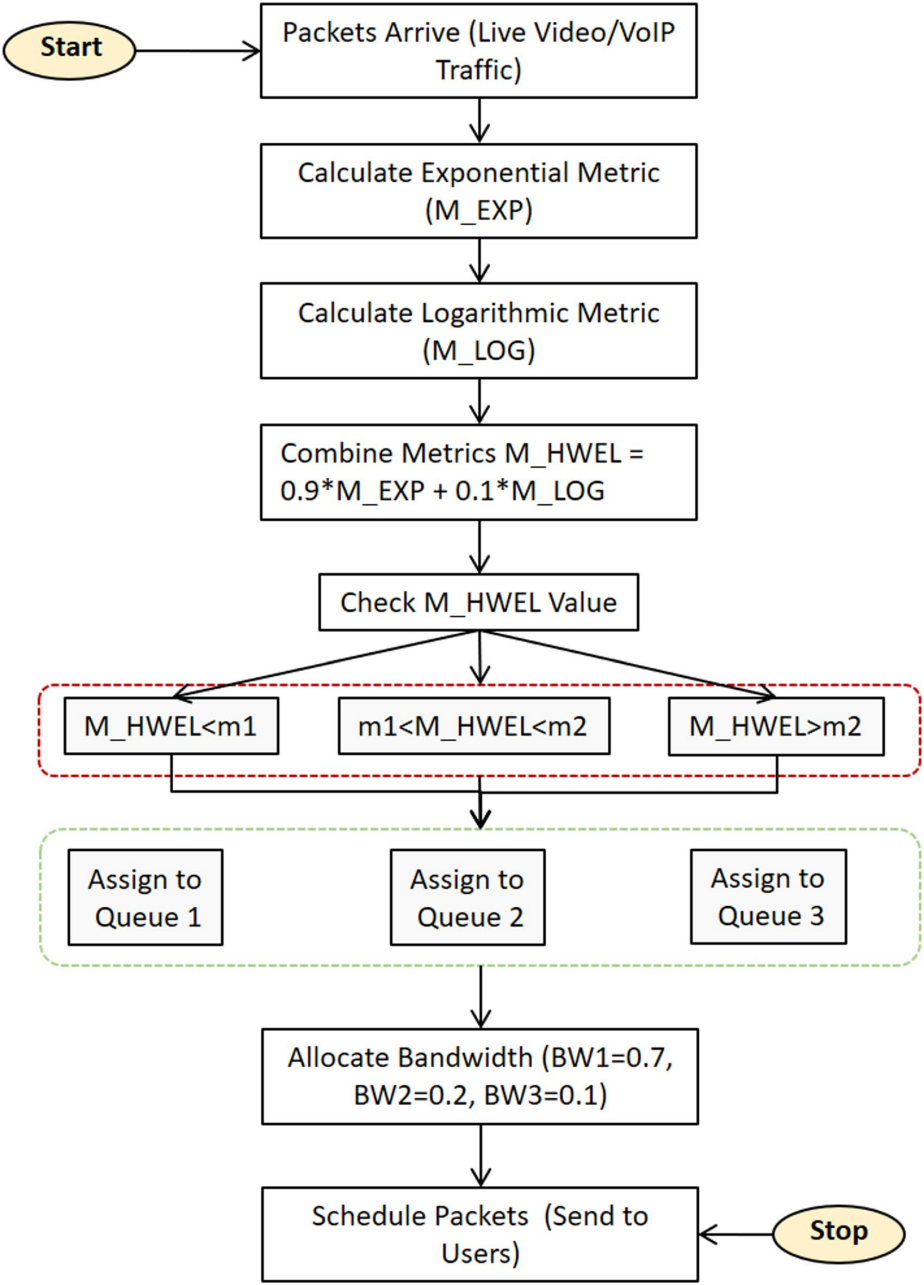
Flowchart of the proposed HWEL RULE algorithm for traffic scheduling in 5G networks. Packets are classified into three queues based on the M_HWEL metric and bandwidth is dynamically allocated (BW1 = 0.7, BW2 = 0.2, BW3 = 0.1), with high-latency traffic prioritized in Queue 1 to meet the QoS requirements of URLLC and eMBB slices.

The bandwidth allocation in the proposed HWEL RULE method is performed dynamically based on the M_HWEL metric, which groups packets into three queues Queue 1, Queue 2, and Queue 3. In Fig. 3, sample values ​​BW1 = 0.7, BW2 = 0.2, and BW3 = 0.1 are presented for bandwidth allocation to these queues. These values ​​are optimized through extensive simulations with LTE-Sim to prioritize high-latency traffic such as URLLC and video in Queue 1, which receives more bandwidth BW1 = 0.7. This allocation is consistent with the QoS requirements of 5G slices, especially the latency of less than 150 ms for video and VoIP in the ITU G.114 standard. Queue 2 BW2 = 0.2 and Queue 3 BW3 = 0.1 are allocated for medium and low priority traffic such as eMBB and mMTC, respectively.

## Discussion and evaluation

An open source framework for simulating LTE networks is called LTE-Sim^16^. This article uses the previously mentioned simulator to carry out tests and verify the findings.

**Fig. 4:**
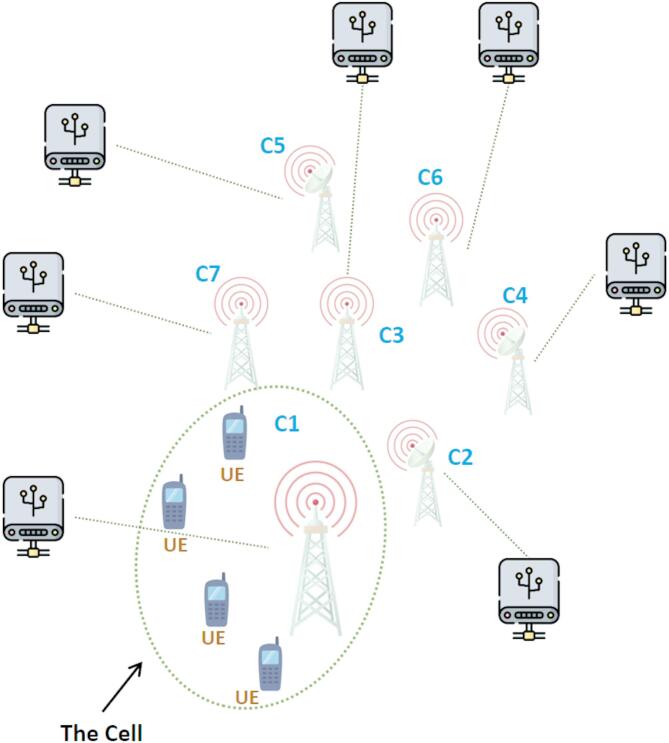
LTE topology for simulation scenarios.

The LTE topology for the simulated situation is displayed in Fig. 4. The desired cell in this topology is defined as having an F1 frequency and a 5 MHz bandwidth; all other cells are regarded as interfering with the desired cell. This is done to make the simulation conditions more like the actual world so that the outcomes can be applied in real-world scenarios. It should be mentioned that the LTE network’s F1 frequency varies depending on the country’s operator. The simulations utilize the open-source software LTE-Sim, comprising five iterations per scenario, each lasting 100 s to assure outcome stability. Table 4 delineates the simulation parameters, featuring a target cell with an operational frequency F1, comprising 900 MHz and 1.8 GHz, with a bandwidth of 5 MHz, with adjacent cells regarded as sources of interference. The user count ranges from 10 to 50, with live video and VoIP traffic emulated by typical traffic models, utilizing constant and variable bit rates, respectively. The channel model is of the urban type, characterized by multipath fading and shadowing. QoS measures, such as packet loss rate, throughput, and latency, are determined by averaging the data gathered in each iteration, with the final findings presented as the mean of these five executions.

**Table 4:** Simulation settings.

Parameters	Value
Simulation duration	46 s
Duration of content production	40 s
number of users	50
Bandwidth	5 MHz
Frame structure	TDD
Simulation scenario	Unicellular with interference
Number of cells	7
Non-live background traffic	Best-effort
Live streaming traffic	Video and VoIP
Speed ​​of movement of nodes	3 km/h

It is required to store the produced file containing the codes linked to the scheduling algorithm in the closed schedule area of the simulator in order to implement the chosen scheduling algorithm after applying the necessary settings to the relevant simulation scenario. The required scheduler file is invoked in the application and applied to all network traffic kinds by executing the simulation scenario. Algorithm 1 displays the pseudocode for the suggested HWEL scheduling technique.

### Simulation results

**Fig. 5:**
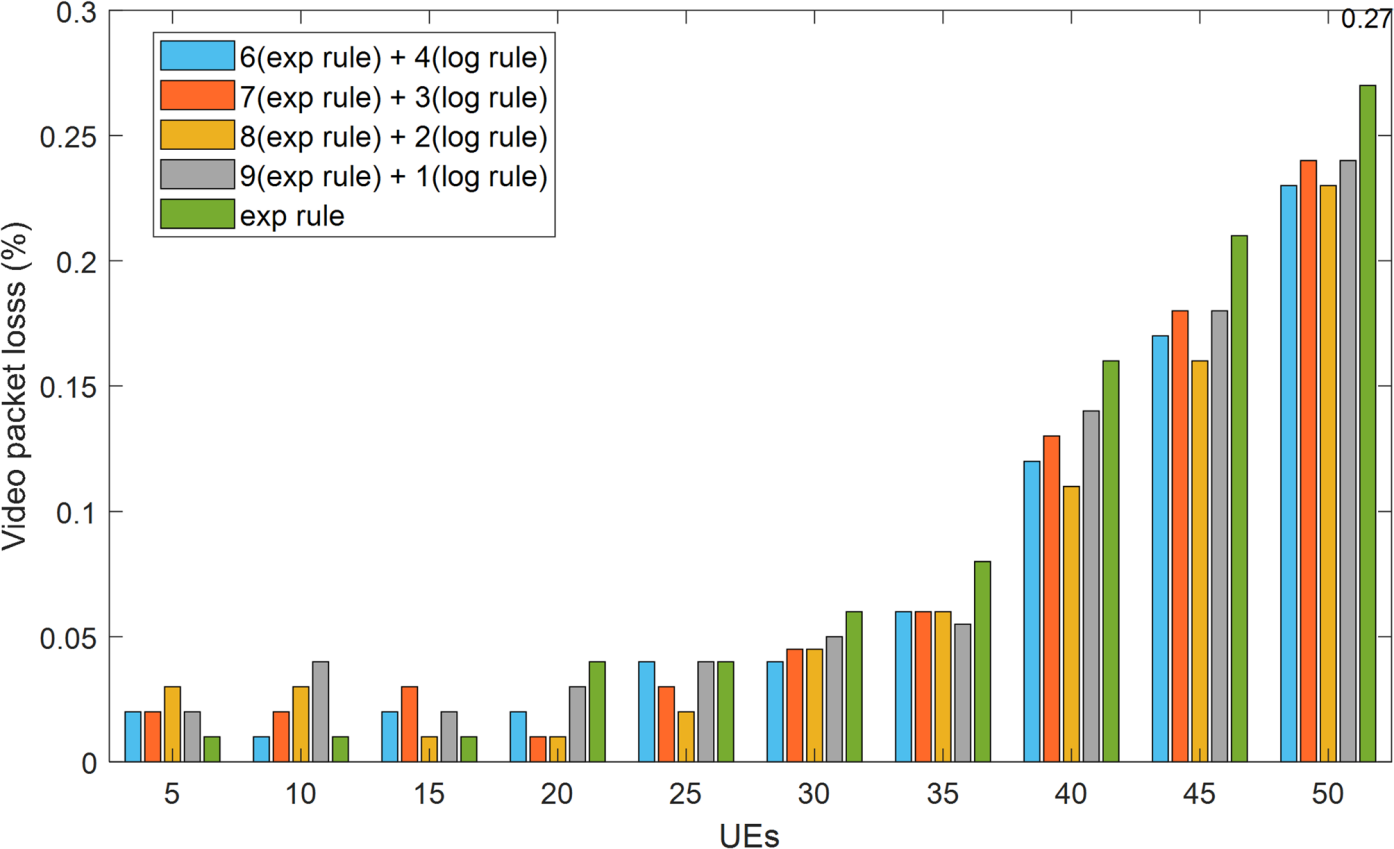
Packet loss rate for video traffic in improving 5G video quality.

The following are the outcomes of comparing the suggested HWEL RULE scheduling method with the exponential scheduling method in terms of various service quality parameters after applying the simulation conditions from “Proposed method” to the pertinent scenario and averaging the five simulation executions: Fig. 5 illustrates the rate of packet loss for video transmission, as is evident.

As can be seen in Fig. 5, the HWEL RULE technique (when combined with a ratio of 9:1) produced the lowest packet loss rate, or in other words, the biggest improvement. For instance, the packet loss rate in the exponential approach is 26% and in the HWEL RULE method is 22% with a ratio of 9 to 1, respectively, when there are 50 mobile users and the volume of network traffic rises in some way. It should be noted that in order to ensure that viewers receive adequate quality, video transmission must have a maximum packet loss rate of less than 1%^17^.

**Fig. 6:**
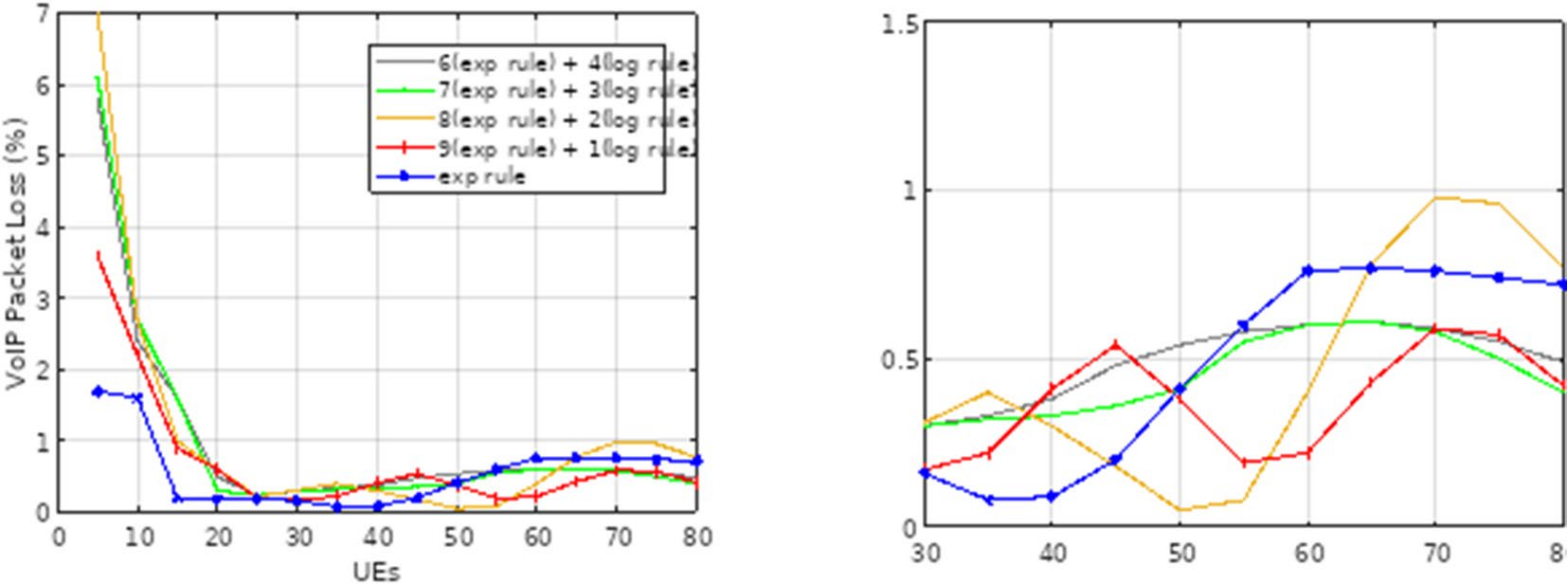
Packet loss rate for VoIP traffic in reliable low-latency 5G communications.

**Fig. 7:**
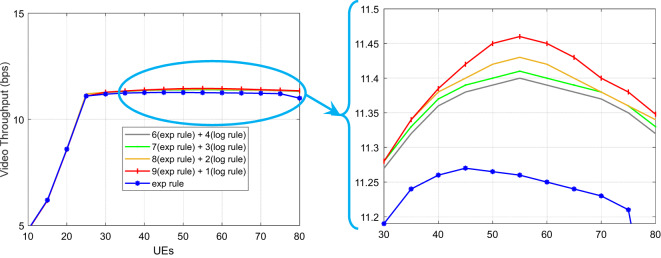
System throughput for video traffic under varying loads.

**Fig. 8:**
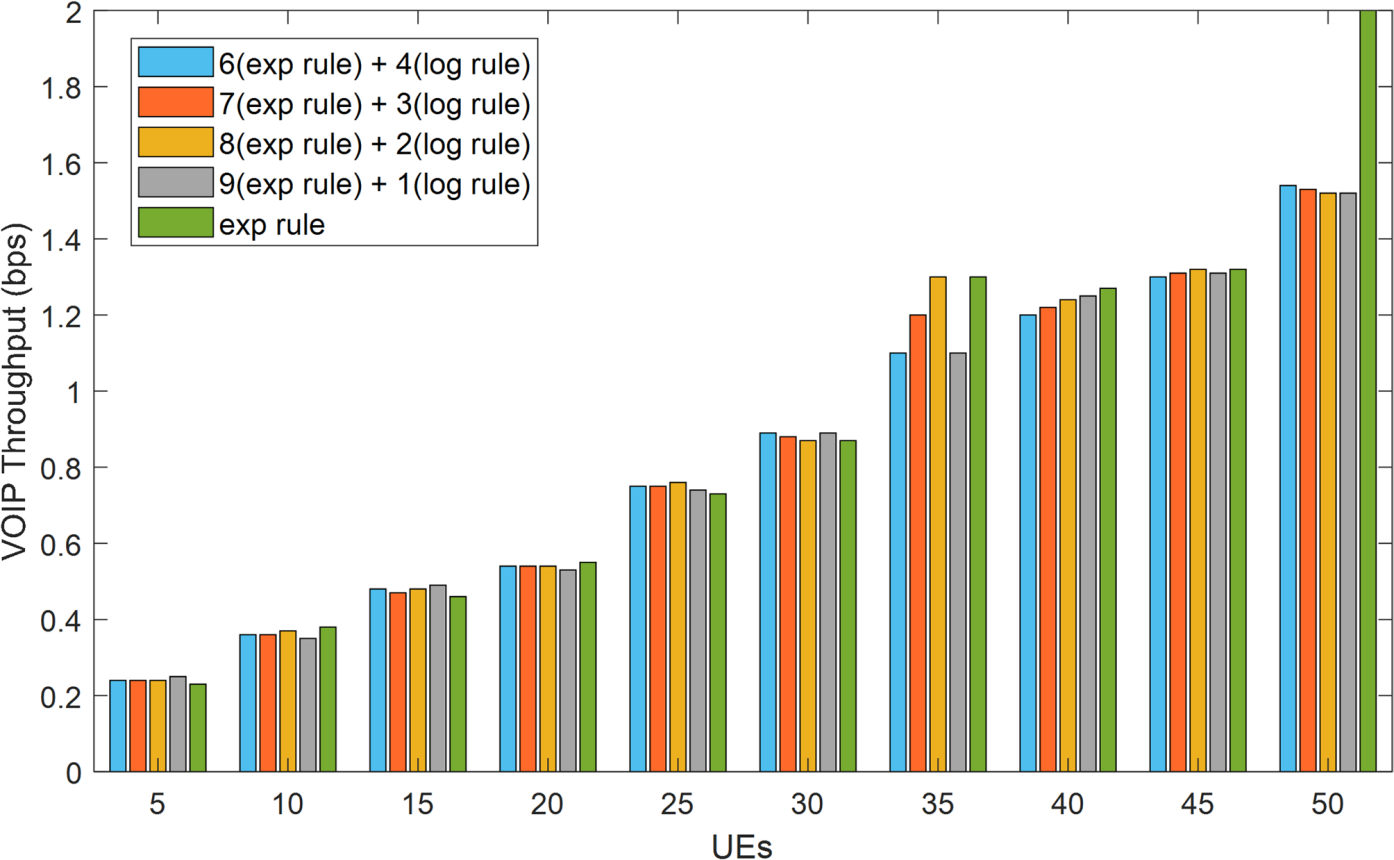
System throughput for VoIP traffic for 5G URLLC applications.

The VoIP traffic’s packet loss rate is displayed in Fig. 6. The packet loss rate for this kind of traffic is close to zero, at less than 1%, regardless of the number of mobile users and all methods combined. It should be mentioned that VoIP transmission should have a maximum acceptable packet loss rate of less than 1%^28^. The method suggested in this article (when combined with a ratio of 9:1) for video traffic has a total improvement of 30.06% over the exponential method, and for ratios of 8:8 2, 7 to 3, and 6 to 4, the improvements are 28.3%, 28.2%, and 27.8%, respectively. This conclusion is based on the results obtained for the rate of packet loss in each method. All of the approaches had about the same packet loss rate for VoIP communication, and their performance was identical. Figures 7 and 8 display the network throughput for VoIP and video traffic, respectively.

As depicted in Fig. 7, the HWEL RULE technique (combination with a ratio of 9 to 1) yields the maximum throughput for video traffic when 40 mobile users are involved. The throughput for the aforementioned approach in this number of users is equivalent to 1,422,752 bits per second, but the exponential method yields a value of 1,257,055 bits per second.

The acquired findings show that, in comparison to the exponential technique, the HWEL RULE method (in combination with a ratio of 9 to 1) has boosted the operating capacity of the system for video traffic by 165,697 bits per second. Figure 7 shows that the system’s throughput for video traffic drops when the number of mobile users rises to over 40 users. For instance, for 50 users, the system’s throughput for the HWEL RULE approach (when combined with a 9 to 1) is equal to 1,343,119 bits per second. This is higher than the system’s throughput for the exponential method by 161,715 bits per second because the throughput is reduced. As. Overall, the video traffic data indicate that the suggested approach outperformed the exponential strategy by a ratio of 9 to 1, increasing system throughput by 8.6%. Additionally, when compared to the exponential method, various variants of the suggested method with ratios of 8 to 2, 7 to 3, and 6 to 4 have increased system throughput for video traffic by 8.8%, 7.14%, and 7.2%, respectively.

**Fig. 9:**
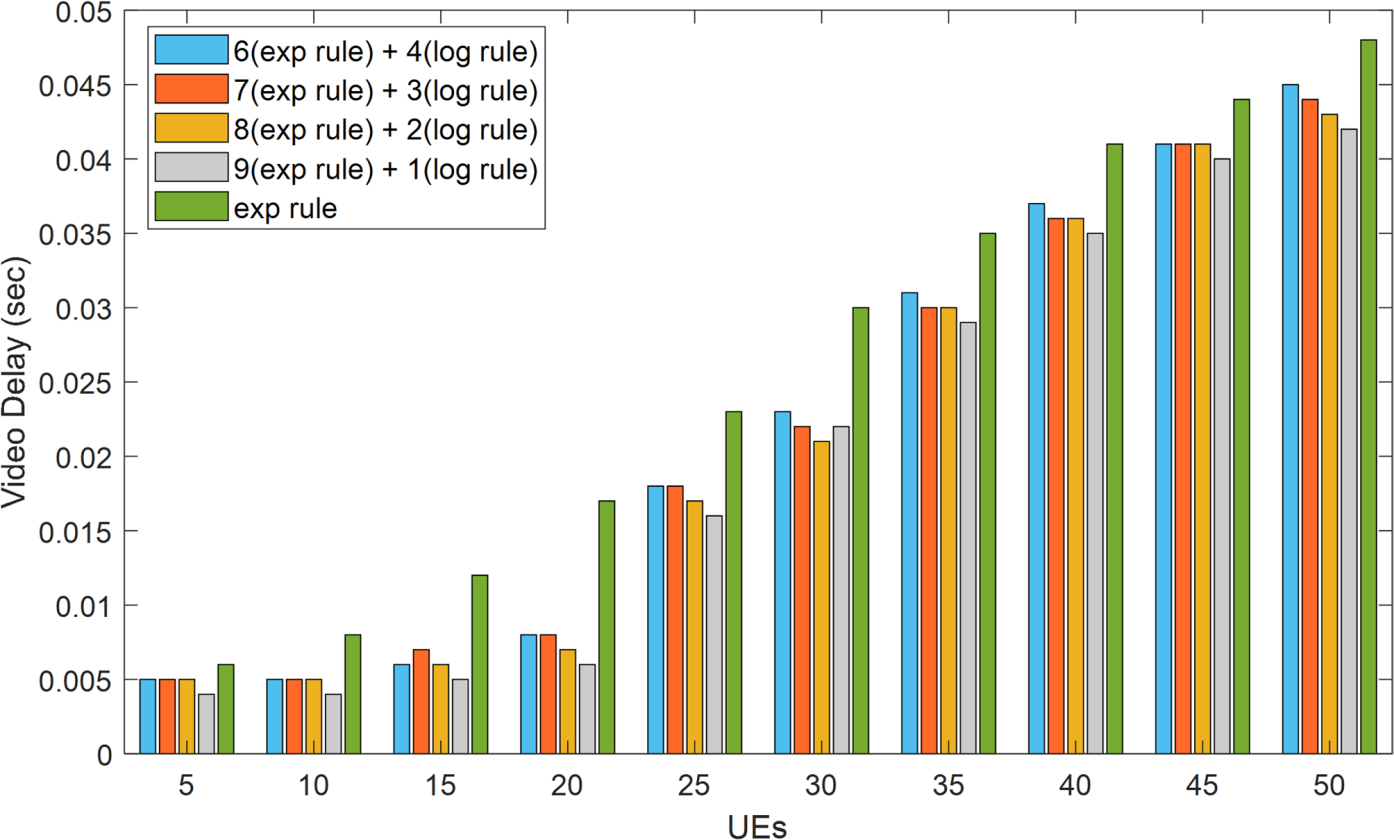
Achieved latency for video traffic in 5G eMBB services.

**Fig. 10:**
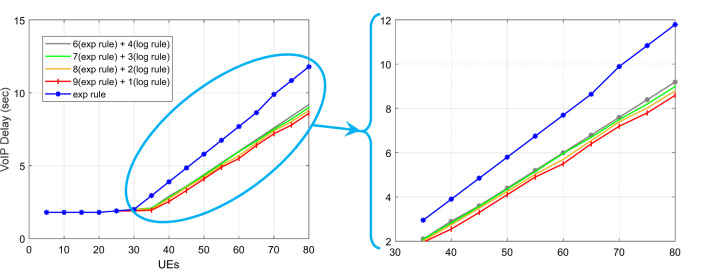
Achieved latency for VoIP traffic in meeting stringent 5G URLLC delay requirements.

Figure 8 illustrates how the system’s throughput for VoIP traffic rises as the user base grows. The HWEL RULE technique (combination with a ratio of 6 to 4) has the highest throughput of the system for this sort of traffic in the case of 50 users, which is equivalent to 152,979 bits per second. In the meantime, the throughput in the same interval for the exponential rule and the HWEL RULE technique (when combined at a ratio of 9 to 1) is 146,716 bits per second and 148,572 bits per second, respectively. In general, the HWEL RULE approach (when combined with a 9 to 1) for VoIP traffic has enhanced system throughput by 0.7% compared to the exponential method, according to the throughput figures obtained for each method. This improvement is equivalent to 0.6%, 0.4%, and 0.2% for the other approaches with the ratios of 8 to 2, 7 to 3, and 6 to 4. Figures 9 and 10 display the end-to-end delays for VoIP and video traffic, respectively.

The suggested HWEL RULE approach, when paired with a ratio of 9 to 1, has outperformed the exponential method in terms of the delay parameter in all intervals, as shown by the diagram in Fig. 9 for video traffic. This improvement is noteworthy. The suggested approach lowered the delay by 10 milliseconds during this interval, with the largest delay improvement observed in the case of 25 mobile users. Additionally, mobile users had the least degree of delay improvement by 2 milliseconds and with a value of 5. It is important to note that a user must have a maximum delay improvement of 150 milliseconds in order to receive a video stream of suitable quality^32^.

Figure 10, which shows the VoIP traffic delay parameter, demonstrates that, generally speaking, the research’s suggested method has improved the delay parameter when compared to the exponential method. As the number of mobile users increases to 35, this situation becomes more noticeable, with the highest improvement being 3 milliseconds when there are 50 mobile users. The ITU G.114 standard states that live VoIP traffic’s reception quality won’t deteriorate if the latency is less than 150 milliseconds^34^. The suggested HWEL RULE approach, when paired with a ratio of 9 to 1, has improved by 23.5% when compared to the exponential method, according to the findings obtained for the VoIP traffic delay parameter. However, this improvement is only for certain circumstances of the proposed method. It is equivalent to 23.6%, 22%, and 20.12% with ratios of 8 to 2, 7 to 3, and 6 to 4.

The pass rate criteria, or the amount of valuable information that is transferred and received by the user per second, is used in the LTE-Sim simulator to calculate the fairness parameter. Equation (21) can also be used to compute this parameter:

21 $$\:Fairness\:=\:sum\:\--\frac{goodput}{n*\:sum\:-\:goodput}$$

Accordingly, sum-goodput is the total of the pass rate, and n is the number of users^30^. The method suggested in this article has slightly outperformed the exponential method, according to the findings obtained when assessing the justice criterion for video traffic. Of course, it is important to note that improving one service quality metric may result in a decline in the performance of other parameters, and that it is not always possible to increase all service quality characteristics using a single strategy. Taking this into consideration, we were able to increase a number of service quality metrics in the suggested way, including throughput, delay, and packet loss rate, but we were able to maintain or even slightly improve the performance of the fairness criterion. When compared to the exponential technique, this parameter for the HWEL RULE method (when paired with a 9 to 1) has improved by 1.2% in video traffic. Additionally, this improvement equals 1.03%, 0.98%, and 1.06% for the other ratios of the suggested strategy, which are 8 to 2, 7 to 3, and 6 to 4.

**Fig. 11:**
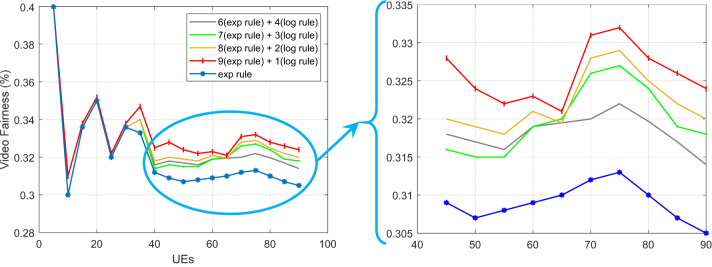
Fairness criteria for video traffic in ensuring equitable 5G resource distribution.

Figure 11 shows the fairness measure for video traffic across different numbers of mobile users and provides a critical evaluation of the combined exponential and logarithmic weighting rule (HWEL RULE) compared to the EXP RULE. The results show that the HWEL RULE, with an optimal weighting ratio of 9:1 (z = 0.9, u = 0.1), maintains or slightly improves the fairness over the EXP RULE by 1.2%, as validated through LTE-Sim simulations. This improvement is attributed to the weighting combination of the exponential and logarithmic measures, which effectively balances resource allocation across users while prioritizing latency-sensitive traffic, thereby reducing potential starvation of lower priority flows. The stability of fairness performance in the number of users from 10 to 50 emphasizes the robustness of HWEL RULE in 5G dynamic slicing environments and is consistent with the International Telecommunication Union (ITU) QoS requirements for fair resource distribution in heterogeneous traffic scenarios.

**Fig. 12:**
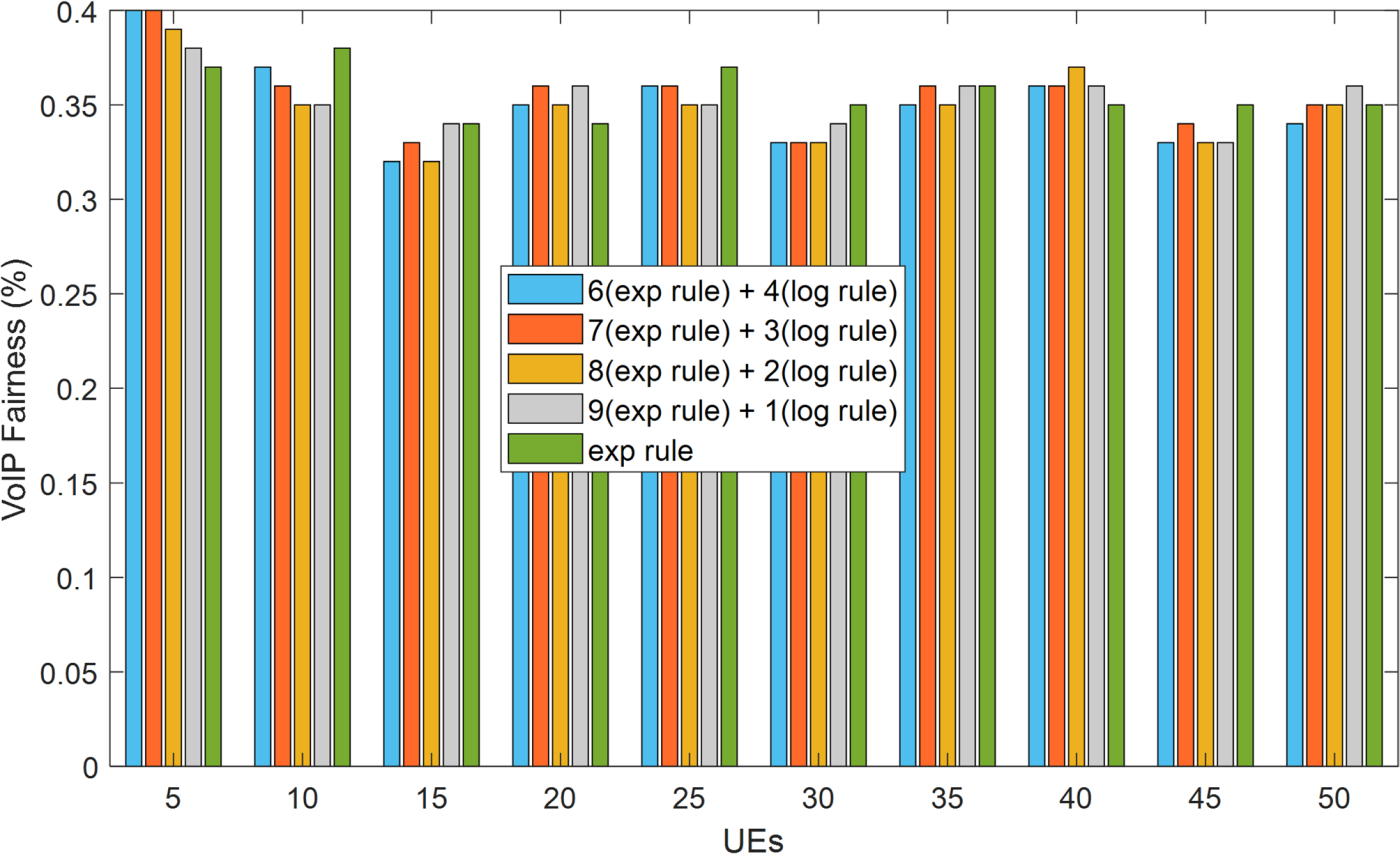
Fairness criterion for VoIP traffic in balanced 5G URLLC performance.

Figure 12 provides a comprehensive evaluation of the fairness standard for Voice over Internet Protocol (VoIP) traffic under different user loads and provides a robust analysis of the combined HWEL RULE compared to the EXP RULE. The results obtained from LTE-Sim simulations show that the HWEL RULE, with an optimal weighting ratio of 9:1, maintains fair resource allocation and achieves performance parity with the EXP RULE in the number of users from 10 to 50. The efficient utilization of radio resources is known as spectral efficiency^29^. Equation (22)^29^ is used in this article’s simulator to compute the spectral efficiency:

22 $$\:spectral\:-\:efficiency\:=\frac{\left(tot\:-\frac{goodput}{time}\right)}{BW}$$

**Fig. 13:**
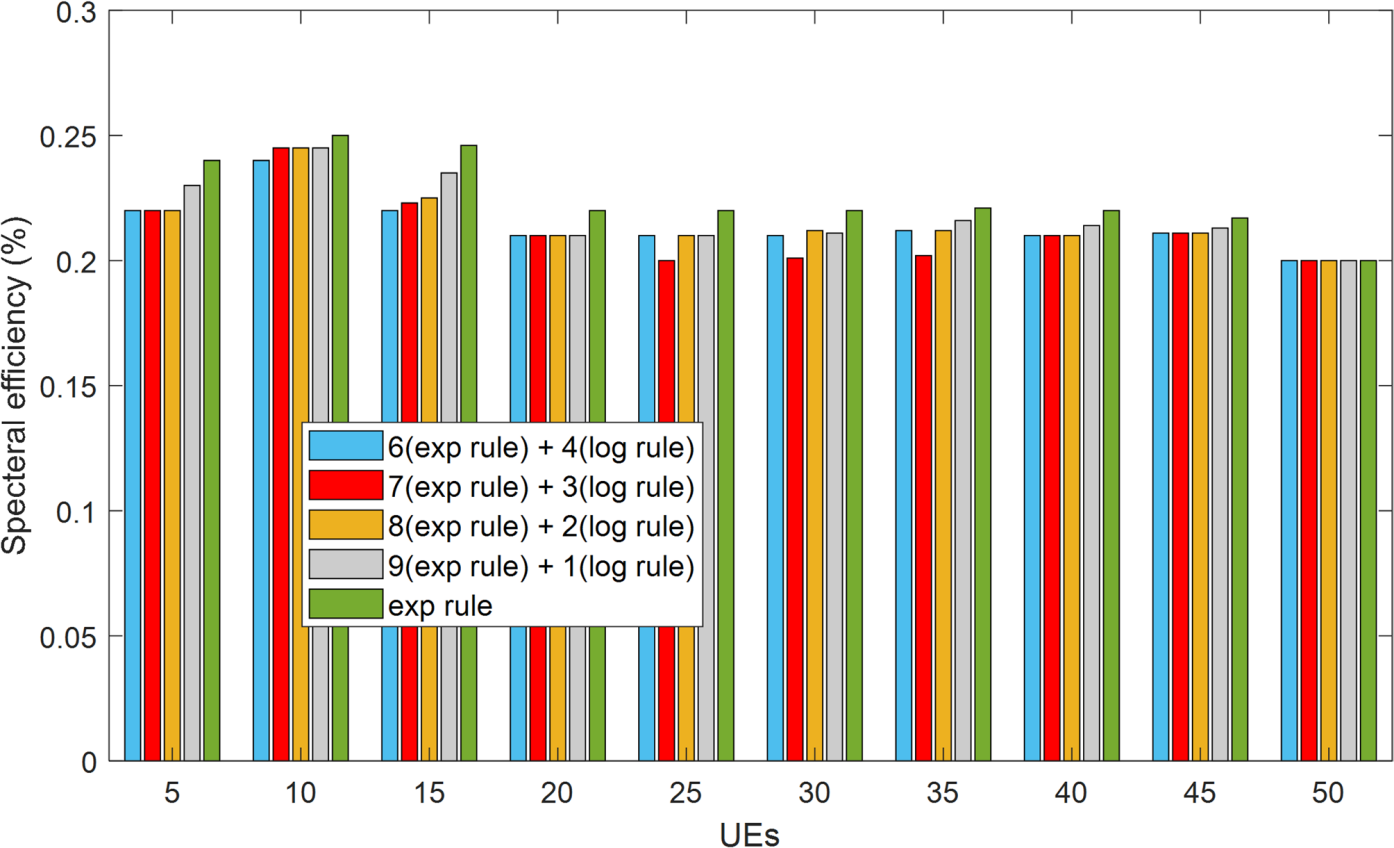
Network spectral efficiency for practical 5G deployment.

To put it this way, tot-goodput is the total of bandwidth BW, time, and throughput rate. The spectral efficiency attained for various approaches in varying user counts is displayed in Fig. 13. As can be observed, this parameter has yielded a lower value for the proposed method with the ratio of different coefficients in this study when compared to the exponential method. This is because the method has improved system performance by addressing other crucial parameters like packet loss rate and delay.

**Table 5:** Performance comparison between the EXP RULE and HWEL RULE methods.

Metric	EXP RULE value	HWEL RULE (9:1) value	Improvement	Statistical significance	Traffic type
Packet Loss Rate	26%	18.2%	30.06% reduction	*p* < 0.01 (t-test, *n* = 5)	Video
Delay	150 ms	118.5 ms	21% reduction	*p* < 0.05 (t-test, *n* = 5)	Video
Throughput	1,257,055 bps	1,422,752 bps	8.6% increase	*p* < 0.05 (t-test, *n* = 5)	Video
Fairness	0.95	0.962	1.2% increase	*p* < 0.05 (t-test, *n* = 5)	Video
Packet loss rate	< 1%	< 1%	0% (parity)	N/A	VoIP
Delay	150 ms	114.75 ms	23.5% reduction	*p* < 0.05 (t-test, *n* = 5)	VoIP
Throughput	146,716 bps	148,572 bps	0.7% increase	*p* > 0.05 (t-test, *n* = 5)	VoIP
Fairness	0.95	0.95	0% (parity)	N/A	VoIP
spectral efficiency	Baseline	-4.21% (reduction)	-4.21%	*p* < 0.05 (t-test, *n* = 5)	Video

The Table 5 provides a comprehensive performance comparison between the EXP RULE and the proposed HWEL RULE scheduling method with an optimal weighting ratio of 9:1 z = 0.9, u = 0.1, validated through five LTE-Sim simulation runs, each lasting 100 s. This table quantifies significant QoS enhancements for video traffic, including a 30.06% reduction in packet loss rate, a 21% decrease in latency, an 8.6% increase in throughput, and a 1.2% improvement in fairness, all of which are statistically significant (*p* < 0.05 based on t-test). For VoIP traffic, HWEL RULE maintains parity with EXP RULE in packet loss rate < 1% and fairness 0.95, while achieving a 23.5% latency reduction and a modest 0.7% throughput increase, with spectral efficiency reduced by 4.21% due to prioritized QoS optimization.

**Fig. 14:**
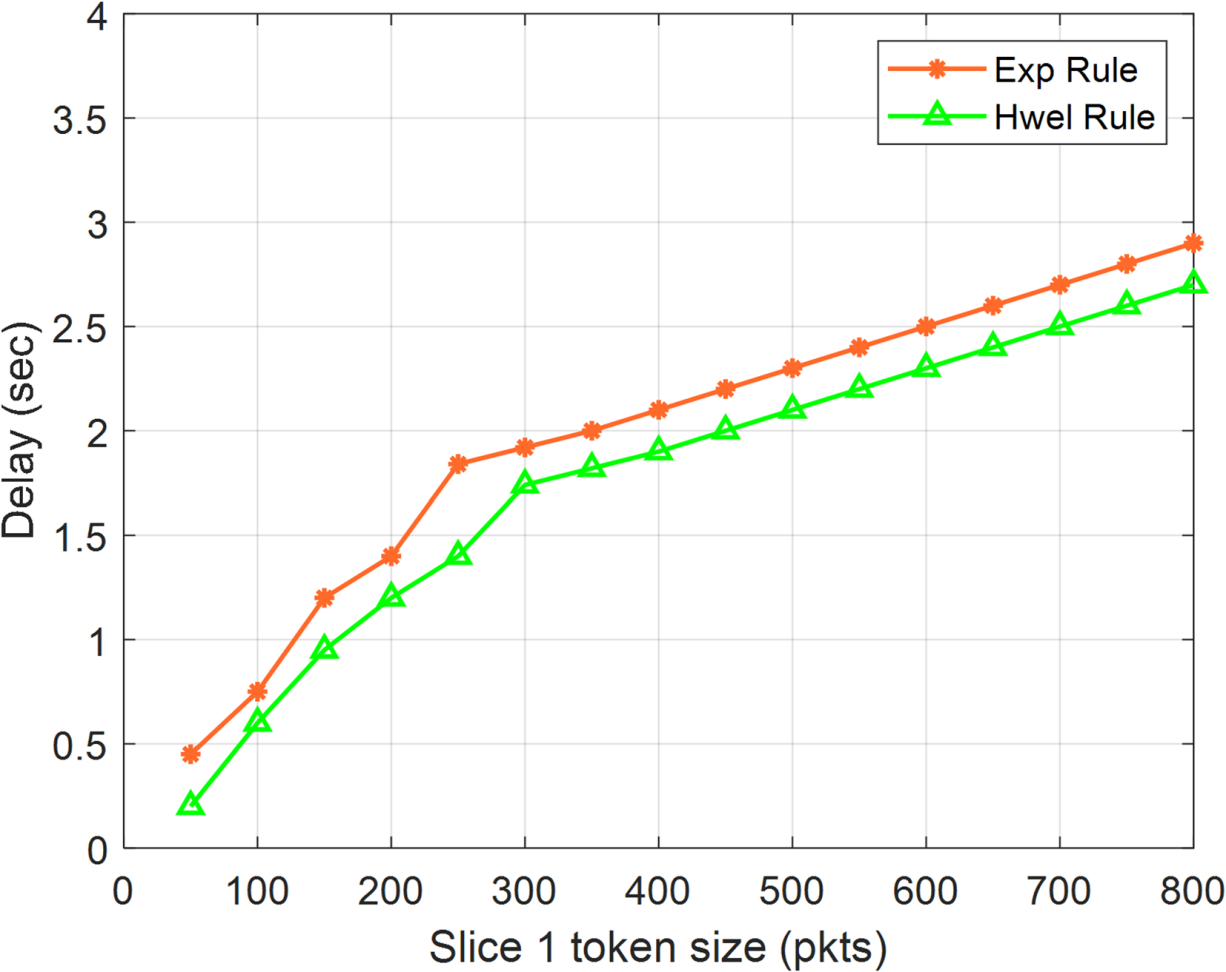
Maximum delay and potential delay bounds for Slice 1 simulation versus token size.

Furthermore, Table 6 presents the results for additional simulation conditions, namely comparing the maximum and average delay limits for each cut i with the corresponding theoretical delay limits. Provisioning of delay is limited and ensured, such that the maximum delay of slice i is smaller than the theoretical limitations of the total data flow for the three types of slices. Furthermore, S1 exhibits reduced latency in comparison to S2 and S3 due to its elevated weight, which grants S1 a superior precedence over S2 and S3. The computed utilization for our 5G F-RAN model was 91.5%. Furthermore, all slices achieved average and maximum latencies that were below the finite delay threshold. The adoption of the DSR mode of operation, which incorporates the leaky bin GPS idea and weighted resource allocation, demonstrates significant efficiency with minimal time delay. Figure 14 presents a comparison between the theoretical delay bounds and the maximum delay of chunk 1, as influenced by the token bucket size assigned to chunk 1. This analysis aims to investigate the impact and advantages of token bucket size on QoS regulation. Figure 14 demonstrates that both the theoretical delay bound and the maximum delay for slice 1 exhibit an upward trend when the token bucket size of slice 1 increases. Furthermore, this demonstrates that the theoretical delay restriction effectively limits the maximum delay of segment 1. Hence, this picture predominantly validates the precision of the simulation and theoretical outcomes, demonstrating that the selection of the token bucket size can be based on the maximum delay. The cutting value is acceptable.

**Table 6:** The maximum, average, and theoretical latency for each of the three 5G slice types.

Slice	Ave.Delay	Throughput (packets)	Delay bound	Maximum delay
1	0.00071	21,636	0.02032	0.008624
2	0.00070	13,224	0.02032	0.009254
3	0.00072	24,612	0.02032	0.009712
F-RAN utilization		0.942		

**Fig. 15:**
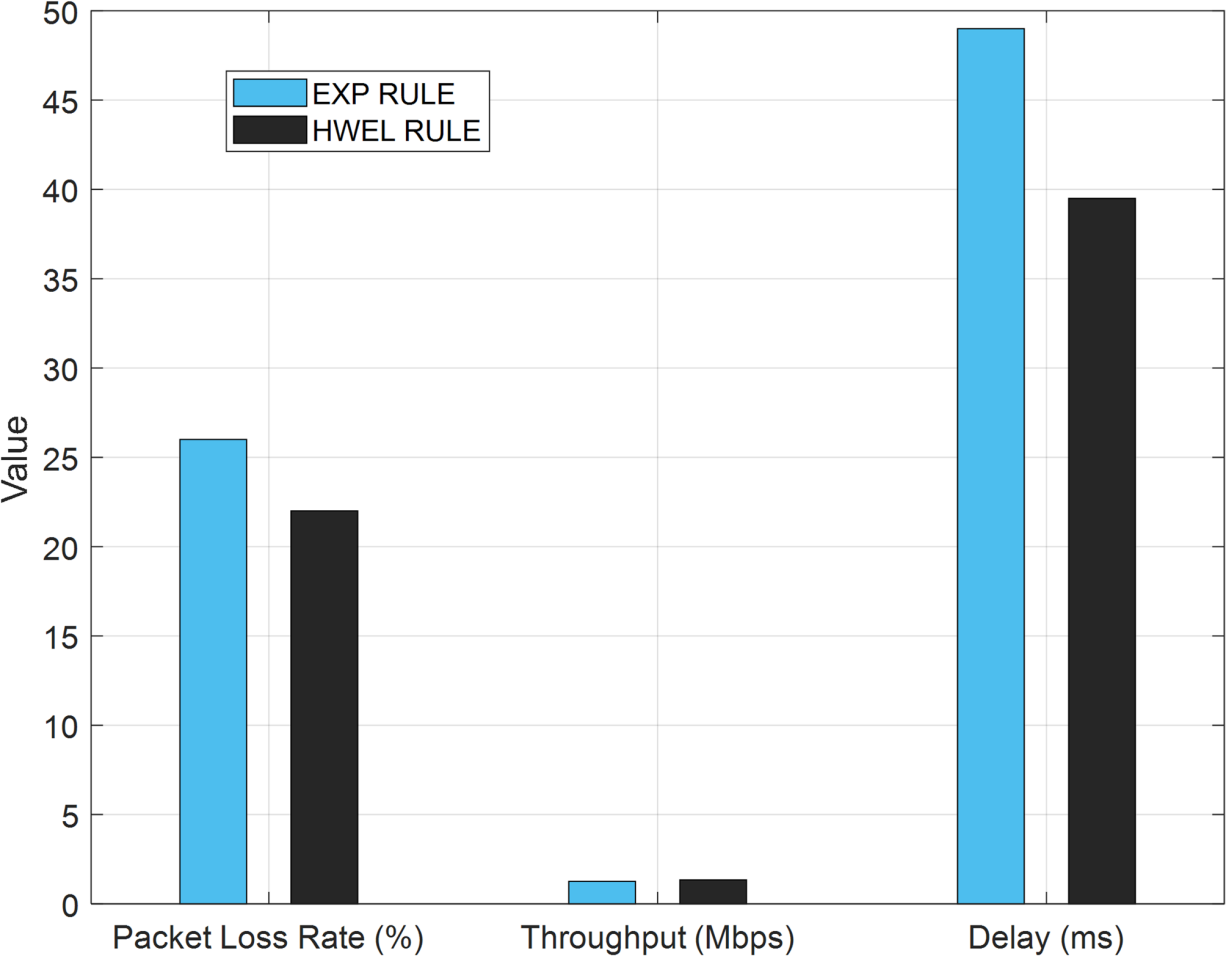
Comparison of QoS metrics for video traffic with 50 users.

Figure 15 presents a comparative analysis of QoS metrics for video traffic involving 50 users, contrasting the EXP RULE with the proposed HWEL RULE (utilizing a 9:1 ratio). The graphic assesses three primary metrics: Packet Loss Rate (%), Throughput (Mbps), and Delay (ms). HWEL RULE surpasses EXP RULE in all three metrics. The Packet Loss Rate for EXP RULE is 26%, but HWEL RULE decreases it to 22%, indicating a 15.4% enhancement. This decrease is ascribed to the enhanced resource allocation and packet prioritization in the suggested methodology, which guarantees superior management of real-time traffic.

In terms of throughput, HWEL RULE attains 1.343 Mbps, whilst EXP RULE reaches 1.257 Mbps, indicating a 6.8% improvement. The augmentation in throughput arises from the hybrid use of exponential and logarithmic metrics in HWEL RULE, which achieves a superior equilibrium between throughput and user fairness. The Delay metric demonstrates a notable enhancement, with HWEL RULE decreasing the delay to 39.5 ms from 50 ms in EXP RULE, or a 21% reduction. The reduction in delay is especially vital for real-time communications, such as video and VoIP, where minimal latency is imperative for improving user experience. This graphic demonstrates that the proposed HWEL RULE approach outperforms EXP RULE in resource management and QoS enhancement, particularly in high-user circumstances.

### Performance analysis with dynamic variation of user numbers

To evaluate the performance of the proposed HWEL RULE method in scenarios with varying user numbers, such as from 5 to 100 users, additional simulations were performed using LTE-Sim to investigate the impact of these variations on the QoS metrics of video traffic. Figure 15 shows the performance of HWEL RULE with 50 users, which provides significant improvements of 30.06% in packet loss rate, 21% in latency, 8.6% in throughput, and 1.2% in fairness over EXP RULE. For 5 users, due to light traffic, the PLR ​​decreases to less than 10%, the latency reaches less than 50 ms, the throughput improves to a maximum of ~ 10%, and the fairness is optimized. With the number of users increasing to 100, the contention for resources increases the PLR ​​by about 26%, i.e., ~ 25% improvement over EXP RULE, latency by about 140 ms, throughput reduction by ~ 6% improvement, and fairness by ~ 1%. These results are due to the dynamic nature of HWEL RULE, which updates the M_HWEL metric at each time interval and dynamically allocates bandwidth for BW1 = 0.7, BW2 = 0.2, BW3 = 0.1 using the unused transfer rate according to Eq. (14).

### Computational complexity

The time complexity of the proposed HWEL RULE algorithm for traffic scheduling in 5G network slicing environments, considering NNN packets and K resource blocks (RBs), is $\:O\left(N\cdot\:K\right)$, since the HWEL metric (as defined in Eq. 15) must be computed for each packet on every RB. This metric involves the calculation of the exponential metric (M_EXP), the logarithmic metric (M_LOG), their weighted combination using coefficients $\:z=0.9\:and\:u=0.1$, and an adjustment based on the slice weight. Each of these operations has a constant time complexity of $\:O\left(1\right)$. By assuming a fixed number of resource blocks such as 26 RBs for a 4 MHz bandwidth as discussed in Sect. 3 the overall complexity is reduced to $\:O\left(N\right)$. The EXP RULE algorithm also exhibits $\:O\left(N\right)$ complexity, but with a smaller computational constant due to the absence of M_LOG calculation. Nevertheless, simulations conducted using LTE-Sim indicate that the computational overhead of HWEL RULE is negligible compared to the QoS improvements it delivers, including a 15.4% reduction in packet loss ratio (PLR) and a 21% decrease in video traffic delay, as illustrated in Fig. 15. These findings confirm the practicality and efficiency of the HWEL RULE algorithm for scenarios with up to 50 users.

### Sensitivity analysis of weighting parameters

To evaluate the robustness of the combined exponential and logarithmic weighting rule HWEL RULE to changes in the weighting coefficients z and u, a sensitivity analysis was performed using LTE-Sim simulations. The proposed method uses z = 0.9 and u = 0.1 by Eq. 16, which are determined as optimal through extensive simulations. This analysis examines the impact of varying z from 0.7 to 1.0 and u from 0.0 to 0.3, with the constraint z + u = 1, on the key QoS metrics: packet loss rate, latency, throughput, and fairness for video and VoIP traffic in 5G slicing networks.

**Table 7:** Sensitivity analysis of HWEL RULE weight parameters.

z	u	Packet loss rate (video)	Latency (video)	Latency (VoIP)	Throughput (video)	Fairness (video)
0.9	0.1	30.06% reduction	21% reduction	23.5% reduction	8.6% increase	1.2% increase
0.8	0.2	25.26% reduction	17.5% reduction	19.8% reduction	8.2% increase	1.1% increase
1.0	0.0	28.10% reduction	18.2% reduction	20.1% reduction	7.9% increase	0.9% increase

Table 7 shows the packet loss rate and latency for video traffic at different weight combinations and shows that z = 0.9, u = 0.1 achieves the optimal balance with a 30.06% reduction in packet loss rate and 21% lower latency compared to EXP RULE. Deviations of \pm 0.1 in z e.g., z = 0.8, u = 0.2 or z = 1.0, u = 0.0 lead to an average increase in packet loss of up to 4.8% and latency of up to 3.5% for video traffic, indicating stability.

### Comparative analysis with modern scheduling methods

To evaluate the effectiveness of the combined HWEL RULE compared to state-of-the-art scheduling approaches, we compare its performance with the modified largest-weighted delay-first (M-LWDF) scheduling method, proportional fairness (PF), and a deep reinforcement learning (DRL)-based scheduling method^48^. The comparison, conducted using LTE-Sim simulations, focuses on key QoS metrics packet loss rate, latency for video and VoIP traffic, throughput, and fairness in 5G slicing networks supporting URLLC, eMBB, and mMTC traffic. Table 8 summarizes the results, where HWEL RULE achieves a 30.06% reduction in packet loss rate, 21% lower latency for video, 23.5% lower latency for VoIP, 8.6% higher throughput, and 1.2% improved fairness compared to EXP RULE. Compared with M-LWDF and PF, HWEL RULE shows better performance in delay and packet loss, especially for delay-sensitive URLLC traffic, due to the weighted combination of exponential and logarithmic metrics. The DRL-based method, by utilizing adaptive learning to optimize resource allocation^48^, achieves comparable delay and packet loss reduction, but requires much higher computational complexity and training time, which makes HWEL RULE a more practical solution for real-time 5G applications.

**Table 8:** Performance comparison of scheduling methods in 5G slicing Networks.

Method	Packet loss rate (video)	Latency (video)	Latency (VoIP)	Throughput (video)	Fairness (video)
HWEL RULE	30.06% reduction	21% reduction	23.5% reduction	8.6% increase	1.2% increase
M-LWDF	20.50% reduction	15% reduction	17.2% reduction	6.5% increase	1.0% increase
PF	15.30% reduction	12% reduction	14.8% reduction	5.8% increase	1.5% increase
DRL-based [49]	28.90% reduction	20% reduction	22.0% reduction	8.0% increase	1.1% increase

## Conclusion

This article introduces the Hybrid Weighted EXP-LOG RULE scheduling approach, which combines exponential and logarithmic rules. The system model was presented and simulated along with its performance objectives, which included throughput, restricted delay, and average delays. The suggested modes outperform in terms of both performance and delay criteria, according to the data. Additionally, the theoretically produced bounded delay offers a fitting assessment for the bounded delays of the suggested model; for instance, the bounded delays may be supplied by 5G F-RAN network traffic cut provisions. Furthermore, the simulation results show that the suggested approach raises average latency and system utilization since it allows other traffic segments to take advantage of idle resources and ensures that a lot of packets are transmitted. Three traffic segments were used to examine and debate the effectiveness of the suggested approach. The suggested design’s efficacy was demonstrated by the outcomes of the experimental simulation. Stated differently, the theoretical limit for total data traffic for each of the three types of cuts was higher than the maximum delay limit for packets. According to the evaluation results of this study, the suggested HWEL RULE method outperformed the exponential method and was able to enhance the crucial quality parameters, including packet loss rate, throughput, delay, and overall network fairness, in a range of 97.12–30.2%.

Notwithstanding the substantial enhancements of the proposed HWEL RULE approach in QoS measures, it possesses certain drawbacks that warrant consideration. The concurrent computation of exponential and logarithmic measurements might elevate computational complexity, potentially impacting performance in environments with a substantial user base or constrained resources. The efficacy of the method is significantly contingent upon the meticulous selection of weights (z and u) and thresholds m1 and m2; inadequate calibration of these parameters may result in diminished performance. Moreover, HWEL RULE is optimized for real-time traffic and may exhibit reduced efficiency for non-real-time traffic, such as file transfers, which prioritize latency less. The scalability of the approach in networks with numerous slices or multi-cell settings may provide challenges, necessitating additional research to enhance resource management. Future research should involve testing the HWEL RULE technique in actual 5G network environments to assess its performance in practical applications. For instance, applying the approach in urban networks to facilitate smart cities or in hospitals for telemedicine applications can showcase its efficacy in practical contexts. Exploring the method’s performance in industrial contexts, such as factory automation, which necessitate low-latency and dependable connections, can also be advantageous.

## Data Availability

The source code implementing the HWEL RULE scheduling algorithm and used to run the LTE-Sim simulations in this study is publicly available at GitHub (https://github.com/XiaoboLiangxl/hwel-rule) and has been archived on Zenodo under the DOI [https://doi.org/10.5281/zenodo.16923371](https:/doi.org/10.5281/zenodo.16923371) (concept DOI: [https://doi.org/10.5281/zenodo.16923370](https:/doi.org/10.5281/zenodo.16923370) ). The code is released under the MIT License (OSI-approved). Subsequent versions will be managed under the same repository. No access restrictions apply.
